# A new microendemic species of the deep-water catshark genus *Bythaelurus* (Carcharhiniformes, Pentanchidae) from the northwestern Indian Ocean, with investigations of its feeding ecology, generic review and identification key

**DOI:** 10.1371/journal.pone.0207887

**Published:** 2018-12-12

**Authors:** Simon Weigmann, Carina Julia Kaschner, Ralf Thiel

**Affiliations:** 1 Elasmo-Lab, Elasmobranch Research Laboratory, Hamburg, Germany; 2 Center of Natural History, University of Hamburg, Hamburg, Germany; 3 Department 522, Fisheries, Federal Office for Agriculture and Food, Hamburg, Germany; University of Minnesota, UNITED STATES

## Abstract

A new deep-water catshark, *Bythaelurus stewarti*, is described based on 121 examined specimens caught on the Error Seamount (Mount Error Guyot) in the northwestern Indian Ocean. The new species differs from all congeners in the restricted distribution, a higher spiral valve turn count and in the morphology of the dermal denticles. It is distinguished from its morphologically and geographically closest congener, *B*. *hispidus* (Alcock), by the larger size (maximum size 44 vs. 39 cm TL, maturity size of males 35–39 vs. 21–28 cm TL), darker fresh coloration and dark grayish-brown mottling of the ventral head (vs. ventral head typically uniformly yellowish or whitish). Furthermore, it has a strongly different morphology of dermal denticles, in particular smaller and less elongate branchial, trunk and lateral caudal denticles that are set much less densely and have a surface that is very strongly and fully structured by reticulations (vs. structured by reticulations only in basal fourth). In addition, the new species differs from *B*. *hispidus* in having more slender claspers that are gradually narrowing to the bluntly pointed tip without knob-like apex (vs. claspers broader and with distinct knob-like apex), more spiral valve turns (11–12 vs. 8–10) and numerous statistical differences in morphometrics. A review of and a key to the species of *Bythaelurus* are given.

## Introduction

The family Scyliorhinidae *sensu lato*, comprising all catsharks of the order Carcharhiniformes, is the largest family of sharks with currently 158 described and valid species (number updated from Weigmann [1,2]). Members of the family reach maximum total lengths from 27 to 162 cm and live in shallow and deep water on continental and insular shelves and slopes in temperate and tropical latitudes of the Indian, Atlantic and Pacific oceans, between depths of 0 to 2200 m [1]. However, the family Scyliorhinidae *sensu lato* has been shown to be paraphyletic and the subfamily Pentanchinae is currently considered a valid family, sister to the family Scyliorhinidae *sensu stricto*. Both families are distinguished by the absence vs. presence of a supraorbital crest [3]. The family Pentanchidae comprises the 11 genera *Apristurus* (39 species), *Asymbolus* (9), *Bythaelurus* (13), *Cephalurus* (1), *Figaro* (2), *Galeus* (18), *Halaelurus* (7), *Haploblepharus* (4), *Holohalaelurus* (5), *Parmaturus* (10), and *Pentanchus* (1), adding up to a total of 109 species (numbers updated from Weigmann [1,2]). The family Scyliorhinidae *sensu stricto* contains the six genera *Atelomycterus* (6 species), *Aulohalaelurus* (2), *Cephaloscyllium* (18), *Poroderma* (2), *Schroederichthys* (5), and *Scyliorhinus* (16), totaling 49 species (numbers updated from Weigmann [1,2]). Species of *Bythaelurus*, originally erected as a subgenus of *Halaelurus* Gill by Compagno [3], are deep-water catsharks with bluntly rounded snouts and soft bodies. In contrast, species of *Halaelurus* live in shallow to moderately deep waters, have pointed snouts, firm bodies, and dorsal eyes and gill openings [3]. *Bythaelurus* species are small to medium-sized catsharks (maximum total lengths from 30 cm to 76 cm) that live in deep water on continental and insular slopes in temperate and tropical latitudes of the Indian and Pacific oceans, between depths of 200 and 1443 m [1,4,5]. However, intrageneric differences in general morphology and body shape, the presence or absence of oral papillae, the presence or absence of a crest of enlarged dermal denticles on the anterior dorsal caudal-fin margin, and, particularly, genetics and reproductive modes have been found [4,5].

A total of four new *Bythaelurus* species have been described from the western Indian Ocean in recent years: *B*. *naylori* Ebert & Clerkin, *B*. *tenuicephalus* Kaschner, Weigmann & Thiel, *B*. *bachi* Weigmann, Ebert, Clerkin, Stehmann & Naylor, and *B*. *vivaldii* Weigmann & Kaschner. Two of these, *B*. *tenuicephalus* and *B*. *bachi*, were described based on specimens collected during cruise 17 of the Russian RV ‘Vityaz’ in 1988/89 as part of one of the largest collections of deep-water chondrichthyans from the western Indian Ocean. In addition to the type specimens of *B*. *tenuicephalus* and *B*. *bachi*, many further *Bythaelurus* specimens were collected during that cruise, including one specimen of *B*. *clevai* (Séret), 523 specimens of *B*. *hispidus* (Alcock), of which 100 specimens were preserved, as well as 180 specimens of another undescribed species, of which 121 were preserved. The undescribed species of *Bythaelurus* is formally described herein based on the 121 specimens. The description represents contribution no. 20 to the series “Deep-water chondrichthyan fishes of RV ‘Vityaz’ cruise 17 and other Soviet cruises in the Indian Ocean”, initiated with the description of *Rhinochimaera africana* [6]. A review of and a key to the species of *Bythaelurus* are given.

## Materials and methods

Institutional acronyms follow Sabaj [7] except for ZMB = Museum für Naturkunde, Leibniz-Institut für Evolutions- und Biodiversitätsforschung, Berlin. Specimens were fixed with formalin and stored in 70% ethanol. Morphometric measurements were taken from all 121 type specimens between perpendicular lines where relevant by vernier caliper to one tenth of a millimeter and largely follow Compagno [8], with total length = TL instead of TOT. In measurements involving the dorsal or ventral origin of the caudal fin, the origin was set far anteriorly including the low anterior fin ridge following Kaschner *et al*. [9], Weigmann *et al*. [4] and Weigmann & Kaschner [5]. Additional measurements after Nakaya *et al*. [10]: precaudal length from snout tip to ventral origin of caudal fin (PRCV), pre-outer nostril length (PONL) from snout tip to a line connecting anterior ends of right and left outer nostrils (equal to Compagno’s [8] prenarial length), pre-inner nostril length (PINL) from snout tip to a line connecting inner ends of right and left inner nostrils, head width at mouth corners (HMCW), interorbital space (INO) between anterior ends of orbits, caudal-fin length (CL) from ventral caudal-fin origin to the tip, caudal-fin height (CH) as greatest height from caudal-fin dorsal margin perpendicularly to apex of the ventral lobe, caudal-fin postventral margin (CPoV) from apex of caudal-fin ventral lobe to subterminal notch, and caudal-fin terminal lobe height (CTH) at subterminal notch. Additional measurements according to Kaschner *et al*. [9]: head width at level of lateral indention of head (slightly before anterior margin of nostrils) (HLIW), head width at level of maximum outer extent of nostrils (HONW) and head width at posterior edge of nostrils (HPNW). Stage of maturity was determined following Stehmann [11]. Terminology of glans clasper components is after Séret [12], Compagno [3], Kaschner *et al*. [9], and Weigmann *et al*. [4]. Vertebral counts and terminology follow Springer & Garrick [13]. Vertebrae and tooth rows were counted from radiographs, vertebrae were counted from all 121 type specimens, tooth rows from a representative selection of 50 specimens. Teeth and dermal denticles were examined using light microscopy and a scanning electron microscope (SEM). The feeding ecology was quantitatively and qualitatively examined from radiographs of all 121 type specimens. Stomach fullness was scored 0 = stomach empty, 1 = stomach partially filled, food remains detectable, 2 = stomach apparently full, extensively filled with food remains. The stomach of one female paratype (295 mm TL, ZMH 26252) was dissected for examination of food remains not identifiable from the radiographs. Tissue samples were taken internally from 10 paratypes of the new species and 14 specimens (13 specimens collected during cruise 17 of RV ‘Vityaz’ plus specimen BMNH 1898.7.13.21) of *B*. *hispidus* for molecular analyses. The catch locations of the 121 type specimens of the new species and verified occurrences of all other species of *Bythaelurus* are shown in the map in the Discussion section, which was generated based on the Global Relief Model ETOPO1 by the NOAA, the National Oceanic and Atmospheric Administration [14]. Country borders, lakes, and rivers were visualized by means of the shapefiles supplied by ESRI for the ArcExplorer-Java Edition for Education 2.3.2 (AEJEE). For a map with all stations of cruise 17 of R.V. ‘Vityaz’ see Weigmann *et al*. [15] or Weigmann *et al*. [16]. None of the specimens analyzed in this study were collected specifically for the purposes of this study. All specimens examined are preserved in museum collections. All type specimens of the new species were caught during cruise 17 of the Russian RV ‘Vityaz’ in 1988 and 1989, an official and authorized expedition exploring the western Indian Ocean from the Gulf of Aden to the southern end of the Madagascar Ridge at Walters Shoals. No special permissions were required to obtain specimens as the areas trawled were not protected.

The type specimens were deposited in the Zoological Museum Hamburg (ZMH).

### Statistical treatment of morphological data

All morphometric and meristic data of the new species were statistically processed, involving ranges, means, and standard deviations. Morphometric and meristic data from 120 type specimens of the new species from the Error Seamount (one paratype with deformed caudal fin was excluded) and from 100 specimens of *B*. *hispidus*, caught off the Socotra Islands (see map in the Discussion section), were used to conduct three discriminant function analyses (DFAs). The DFAs were performed to determine if these two morphologically and geographically closest *Bythaelurus* species could be differentiated based on morphological parameters using XLSTAT (version 19.7.48622, 13.12.2017, Addinsoft), a statistical analysis add-in for Microsoft Excel.

DFA was used to demonstrate the degree of separation in multivariate space defined by the main patterns of morphological variation among species, which is described via the discriminant functions. It also shows which character contributes more to the differentiation. The standardized discriminant function coefficients represent the contributions of every variable to the discriminatory power of the function. Hence, the larger the standardized coefficient, the larger the weight of the variable in the function.

The first discriminant analysis was conducted for 120 individuals of the new *Bythaelurus* species and 100 specimens of *B*. *hispidus*. The second discriminant analysis was performed for 57 males and 63 females of the new species and 70 males and 30 females of *B*. *hispidus*. The third discriminant analysis was conducted for 7 and 42 adult males of the new species and of *B*. *hispidus*, respectively. Morphological variables with multicolinearity, variables including other variables, and variables not available for all specimens were excluded from the DFA procedures. The first two DFAs were performed for the following 67 morphological characters (for abbreviations see Table 1 in the Results section): PRVC, HDL, PG1, PSP, POB, PP1, PP2, IDS, DCS, PPS, PAS, ACS, PCA, PONL, POR, EYL, EYH, ING, GS2, GS3, GS4, GS5, P1B, P1I, P1P, P1L, D1L, D1B, D1I, D1P, D2A, D2H, D2I, D2P, P2A, P2B, P2H, P2I, ANA, ANH, ANI, ANP, CH, CPrV, CPoV, CTH, CTL, TRH, TAH, CPH, MOL, MOW, ULA, LLA, NOW, INW, ANF, INO, SPL, ESL, HDW, TRW, TAW, CPW, trunk vertebral centra, total precaudal centra, and caudal centra. The third DFA was conducted for the following four morphometric parameters: TL, CLO, CLI, and CLB.

**Table 1:** *Bythaelurus stewarti* n. sp., morphometrics and meristics. Individual values for the adult male holotype (ZMH 26251) and one gravid female paratype (ZMH 26253), ranges for all other paratypes (n = 119 for the meristics except for n = 48 for the tooth row counts, but n = 118 for the morphometrics because the measurements of one paratype were excluded due to its deformed caudal fin), as well as means and standard deviations (SD) for all 121 type specimens concerning the meristics except for n = 50 for the tooth row counts, but for 120 type specimens concerning the morphometrics due to exclusion of the measurements of one paratype with deformed caudal fin. Proportional values are expressed as percentages of total length (TL) 70% ethanol preserved except for minimum, maximum, and mean of TL in mm.

	*B*. *stewarti* n. sp. adult male holotype ZMH 26251		*B*. *stewarti* n. sp. gravid female paratype ZMH 26253		Minimum	Maximum	Mean	SD
	mm	% TL	mm	% TL	% TL	% TL	% TL	
TL, total length	425.0	100.0	425.0	100.0	137.3	417.0	244.8	
PRC, precaudal length dorsally [8]	314.0	73.9	310.0	72.9	67.6	73.0	70.4	1.7
PRVC, precaudal length ventrally [10]	303.5	71.4	305.0	71.8	66.1	71.8	68.8	1.4
PD2, pre-D2-length	273.9	64.4	276.9	65.1	57.5	64.8	61.5	1.4
PD1, pre-D1-length	198.4	46.7	202.3	47.6	41.8	47.1	44.6	1.1
HDL_1, head length (to middle end of fifth gill slit)	89.7	21.1	92.9	21.8	19.8	23.5	22.0	0.7
HDL_2, head length (to level upper end of fifth gill slit)	90.9	21.4	93.8	22.1	19.6	24.0	22.1	0.8
PG1, prebranchial length	69.1	16.2	73.5	17.3	14.8	19.3	17.1	0.7
PSP, prespiracular length	41.8	9.8	44.8	10.5	8.9	12.5	10.7	0.6
POB, preorbital length	23.5	5.5	26.0	6.1	4.9	7.4	6.1	0.5
PP1, prepectoral length	84.6	19.9	83.8	19.7	18.0	22.6	20.3	0.9
PP2, prepelvic length	177.5	41.8	183.1	43.1	38.4	43.5	41.1	1.1
SVL, snout—anterior vent length	191.9	45.1	190.6	44.8	39.6	45.8	43.2	1.2
PAL, pre-anal fin length	256.0	60.2	255.6	60.1	51.4	59.9	56.5	1.6
IDS, interdorsal space	52.1	12.3	49.8	11.7	8.2	13.1	10.9	0.9
DCS, dorsal (D2)—caudal space	13.7	3.2	11.4	2.7	0.0	5.4	1.8	1.1
PPS, pectoral—pelvic space	70.9	16.7	77.1	18.1	13.3	19.7	16.2	1.3
PAS, pelvic—anal space	53.3	12.6	46.9	11.0	8.0	12.7	10.1	1.1
ACS, anal—caudal space	7.5	1.8	6.1	1.4	0.0	1.9	0.6	0.6
PCA, pelvic—caudal space	101.1	23.8	96.8	22.8	20.0	28.8	22.3	1.2
VCL, anterior vent—caudal tip length	228.4	53.7	233.4	54.9	52.9	60.9	56.3	1.3
PONL, pre-outer nostril length	12.5	2.9	13.3	3.1	1.7	3.7	3.0	0.3
PINL, pre-inner nostril length	19.9	4.7	22.0	5.2	3.5	5.7	4.9	0.4
POR, preoral length	24.7	5.8	24.3	5.7	4.5	6.7	5.8	0.4
EYL, eye length	14.5	3.4	14.2	3.3	2.9	4.5	3.5	0.3
EYH, eye height	7.0	1.6	7.7	1.8	1.4	3.0	2.2	0.3
ING, intergill length 1st to last slit (upper end to upper end)	20.6	4.8	20.7	4.9	3.8	7.1	5.3	0.7
GS1, 1st gill slit height (unspread)	6.6	1.5	6.8	1.6	0.7	2.5	1.6	0.3
GS2, 2nd gill slit height	7.1	1.7	7.1	1.7	0.9	2.8	1.9	0.4
GS3, 3rd gill slit height	8.9	2.1	8.5	2.0	1.0	2.9	2.1	0.4
GS4, 4th gill slit height	9.9	2.3	6.2	1.4	0.9	3.0	2.1	0.4
GS5, 5th gill slit height	6.7	1.6	3.2	0.8	0.7	2.3	1.5	0.3
P1A, pectoral anterior margin length	54.0	12.7	54.4	12.8	9.0	14.8	11.8	1.0
P1B, pectoral base length	23.3	5.5	27.3	6.4	3.2	6.7	5.5	0.6
P1I, pectoral inner margin length	22.8	5.4	25.5	6.0	3.3	8.3	5.9	0.9
P1P, pectoral posterior margin length	36.3	8.5	39.4	9.3	4.1	11.1	7.9	1.4
P1H, pectoral height (base end to tip)	42.8	10.1	46.5	10.9	7.0	12.4	9.9	1.2
P1L, P length (ant. base to post. tip)	47.4	11.2	47.9	11.3	8.8	12.1	10.7	0.7
D1L, D1 total length	34.7	8.2	38.1	9.0	7.8	10.7	9.0	0.5
D1A, D1 anterior margin length	37.1	8.7	40.8	9.6	7.4	10.5	9.2	0.6
D1B, D1 base length	26.3	6.2	28.5	6.7	5.0	7.2	6.1	0.5
D1H, D1 vertical height	17.7	4.2	23.0	5.4	2.3	6.1	4.0	0.7
D1I, D1 inner margin length	8.7	2.0	9.6	2.3	1.4	4.4	2.4	0.4
D1P, D1 posterior margin length	15.2	3.6	20.4	4.8	1.5	4.7	3.3	0.6
D2L, D2 total length	32.9	7.7	35.9	8.5	7.0	11.2	8.7	0.7
D2A, D2 anterior margin length	32.4	7.6	36.3	8.5	7.0	11.5	8.7	0.7
D2B, D2 base length	26.4	6.2	27.2	6.4	5.1	8.9	6.5	0.6
D2H, D2 vertical height	12.2	2.9	15.2	3.6	1.8	4.1	2.8	0.5
D2I, D2 inner margin length	7.2	1.7	9.1	2.1	0.9	2.9	1.9	0.3
D2P, D2 posterior margin length	10.0	2.4	13.0	3.1	1.4	3.3	2.5	0.4
P2L, pelvic total length	39.7	9.4	41.4	9.7	7.0	10.8	9.1	0.8
P2A, pelvic anterior margin length	24.3	5.7	21.5	5.0	3.3	6.8	5.6	0.6
P2B, pelvic base length	24.1	5.7	26.0	6.1	3.3	7.4	5.4	0.7
P2H, pelvic height = max. width (excl. clasper)	12.0	2.8	16.7	3.9	1.3	5.2	3.4	0.6
P2I, pelvic inner margin length	15.4	3.6	14.8	3.5	1.8	5.4	3.5	0.6
P2P, pelvic posterior margin length	21.5	5.1	27.1	6.4	3.2	6.7	5.0	0.7
ANL, anal fin total length	50.1	11.8	55.7	13.1	10.9	17.2	13.1	0.8
ANA, anal fin anterior margin length	29.8	7.0	35.2	8.3	5.2	13.3	8.8	1.0
ANB, anal fin base length	43.6	10.3	50.0	11.8	8.9	15.4	11.7	0.9
ANH, anal fin vertical height	13.3	3.1	15.7	3.7	2.0	4.4	3.2	0.5
ANI, anal fin inner margin length	6.6	1.5	7.0	1.6	0.8	2.0	1.4	0.3
ANP, anal fin posterior margin length	25.4	6.0	29.0	6.8	4.5	10.2	6.3	0.7
CL, caudal fin length	120.4	28.3	120.7	28.4	28.2	34.4	30.6	1.2
CH, caudal fin height	24.3	5.7	27.4	6.4	5.3	7.3	6.3	0.4
CPrV, caudal fin preventral margin	44.8	10.5	41.7	9.8	8.8	14.8	11.1	1.0
CPoV, caudal fin postventral margin	54.3	12.8	49.9	11.7	10.6	15.4	12.9	0.8
CTH, caudal fin terminal lobe height	11.3	2.7	12.9	3.0	2.1	3.7	3.0	0.3
CTL, caudal fin terminal lobe length	21.7	5.1	28.3	6.7	3.6	7.8	6.5	0.6
HDH, head height at P origin	31.6	7.4	34.4	8.1	4.5	9.7	6.9	0.9
TRH, trunk height at P base end	34.3	8.1	39.1	9.2	4.8	11.4	7.5	1.1
ABH, abdomen height at D1 base end	21.2	5.0	25.3	6.0	4.4	6.1	5.4	0.3
TAH, tail height at pelvic base end	21.2	5.0	24.3	5.7	4.3	6.0	5.1	0.4
CPH, caudal peduncle height at C origin	13.0	3.1	13.6	3.2	2.8	3.8	3.4	0.2
MOL, mouth length (arc radius)	14.7	3.4	15.2	3.6	2.6	4.8	3.6	0.4
MOW, mouth width	35.6	8.4	34.0	8.0	4.6	8.8	7.6	0.6
ULA, upper labial furrow length	5.4	1.3	3.9	0.9	0.7	1.6	1.1	0.2
LLA, lower labial furrow length	6.4	1.5	6.0	1.4	1.0	2.3	1.6	0.2
NOW, nostril width	12.0	2.8	13.2	3.1	2.2	4.1	3.2	0.3
INW, internarial width	12.6	3.0	12.7	3.0	2.4	4.1	3.0	0.3
ANF, anterior nasal flap length	4.8	1.1	4.0	0.9	0.7	1.5	1.1	0.2
INO, interorbital space at ant. orbits	27.3	6.4	27.0	6.4	6.1	8.0	7.0	0.3
SPL, spiracle length	3.4	0.8	2.9	0.7	0.2	1.2	0.6	0.2
ESL, eye—spiracle length	0.0	0.0	1.8	0.4	0.0	1.3	0.5	0.3
HLIW, head width at level of lateral indention	29.1	6.9	30.1	7.1	6.4	8.0	7.4	0.3
HONW, head width at max. outer extent of nostrils	34.5	8.1	36.5	8.6	8.2	10.1	8.9	0.5
HPNW, head width at posterior edge of nostrils	39.6	9.3	40.7	9.6	9.3	11.0	10.0	0.5
HMCW, head width at mouth corners	51.2	12.0	51.5	12.1	10.3	13.3	11.8	0.6
HDW, head width at 3rd gill slits	48.4	11.4	47.8	11.2	7.5	11.9	9.8	0.9
TRW, trunk width at P base ends	44.9	10.6	48.5	11.4	5.7	11.4	8.6	1.1
ABW, abdomen width at D1 base end	17.1	4.0	19.4	4.6	3.0	4.6	3.9	0.3
TAW, tail width at pelvic base ends	18.3	4.3	20.4	4.8	3.3	5.1	4.4	0.3
CPW, C peduncle width at C origin	8.6	2.0	11.0	2.6	1.8	2.9	2.3	0.2
CLO, clasper outer margin length	26.8	6.3	-	-	1.3	8.5	2.9	1.7
CLI, clasper inner margin length	43.0	10.1	-	-	2.7	11.3	5.5	2.1
CLB, clasper base width	6.1	1.4	-	-	0.5	1.5	0.8	0.2
trunk vertebral centra	38		39		37	42	38.5	0.9
diplospondylous precaudal centra	43		44		37	45	41.0	1.7
total precaudal centra	81		83		75	84	79.5	1.8
caudal centra	55		55		44	59	52.6	3.2
total centra	136		138		125	140	132.1	3.3
tooth rows in upper jaw (approximately)	77		80		64	85	74.8	5.6
tooth rows in lower jaw (approximately)	74		78		64	88	75.6	6.1

### Nomenclatural acts

The electronic edition of this article conforms to the requirements of the amended International Code of Zoological Nomenclature, and hence the new names contained herein are available under that Code from the electronic edition of this article. This published work and the nomenclatural acts it contains have been registered in ZooBank, the online registration system for the ICZN. The ZooBank LSIDs (Life Science Identifiers) can be resolved and the associated information viewed through any standard web browser by appending the LSID to the prefix “http://zoobank.org/”. The LSID for this publication is: urn:lsid:zoobank.org:pub:B24567BF-F872-4059-99EB-7EF32E1306A2. The electronic edition of this work was published in a journal with an ISSN, and has been archived and is available from the following digital repositories: PubMed Central, LOCKSS.

## Results

### Systematic account

***Bythaelurus stewarti*** Weigmann, Kaschner & Thiel **n. sp.** urn:lsid:zoobank.org:act:3E649CDA-2441-4DFF-90FE-8A731948E5EC

Error Seamount Catshark

Holotype **ZMH 26251**, adult male, 435 mm TL fresh, 425 mm TL 70% ethanol preserved, RV ‘Vityaz’, cruise 17, station 2825, Error Seamount (Mount Error Guyot), 10°19’30” N, 56°08’48” E– 10°18’54” N, 56°06’42” E, 395–420 m depth, 28 m shrimp trawl, trawl # 98, on the bottom for 54 minutes, 14 Jan 1989.

Paratypes (120) **ZMH 26252**, 70 specimens: 40 juvenile females, 168–323 mm TL in fresh state, 164–315 mm TL 70% ethanol preserved, 30 juvenile males, 164–348 mm TL in fresh state, 157–340 mm TL 70% ethanol preserved, RV ‘Vityaz’, cruise 17, station 2573, Error Seamount, 10°18’5” N, 56°07’3” E– 10°06’3” N, 56°07’0” E, 408 m depth, 29 m shrimp trawl, trawl # 8, on the bottom for 62 minutes, 30 Oct 1988 (taken together with 43 further specimens: 22 females and 21 juvenile males, which were not retained); **ZMH 26253**, 3 specimens: gravid female, 437 mm TL fresh, 425 mm TL 70% ethanol preserved, with two female embryos, 137.3 and 145.3 mm TL 70% ethanol preserved, RV ‘Vityaz’, cruise 17, station 2575, Error Seamount, 10°17’8” N, 56°06’1” E– 10°22’8” N, 56°06’2” E, 380–400 m depth, Isaacs-Kidd midwater trawl in 400–1100 m depth but with bottom contact in 380–400 m depth, 30 Oct 1988; **ZMH 26254**, 47 specimens: 20 juvenile females, 156–328 mm TL in fresh state, 149.3–315 mm TL 70% ethanol preserved, 20 juvenile males, 162–322 mm TL in fresh state, 147.1–316 mm TL 70% ethanol preserved, 1 possibly subadult female, 369 mm TL in fresh state, 355 mm TL 70% ethanol preserved, 6 adult males, 389–430 mm TL in fresh state, 375–417 mm TL 70% ethanol preserved, data the same as holotype ZMH 26251 (taken together with 16 further specimens: 15 juvenile females and 1 juvenile male, which were not retained). All 121 preserved and 59 discarded specimens were collected by M.F.W. Stehmann (ICHTHYS).

#### Diagnosis

A medium-sized *Bythaelurus* species with the following characteristics: body firm and slender; snout long (preorbital length 4.9–7.4% TL) and broad, bell-shaped in dorsoventral view with distinct lateral indention; pre-outer nostril length 0.6–1.4 times internarial space; preorbital snout length 0.7–1.1 times interorbital space; preoral snout length 0.8–1.7 times in mouth width; eye length 10.2–15.5 times in predorsal distance, 4.9–7.7 times in head length and 1.2–2.3 times eye height; head length 2.2–2.6 times width at level of maximum outer extent of anterior nostrils; head width at level of maximum outer extent of anterior nostrils 1.1–1.3 times width at level of lateral indention of head, 1.2–1.6 times preorbital length, and 8.1–10.1% TL; tongue and roof of mouth densely set with knob-like oral papillae; pelvic-fin anterior margin 1.6–3.5 times in pectoral-fin anterior margin; first dorsal-fin base 1.3–2.3 times in interdorsal space; length of second dorsal-fin inner margin 0.8–2.3 times in second dorsal-fin height; second dorsal-fin base length 5.1–8.9% TL; anal-fin base 0.7–1.9 times interdorsal space. Coloration: dorsally dark grayish-brown with rather indistinct dark blotches at nape, on flank, below both dorsal fins, and across caudal fin; ventral side grayish-white, usually with dark grayish-brown mottling on head. Upper jaw with 64–85 and lower jaw with 64–88 rows of small tricuspidate teeth with outer surface of crown furrowed by strong longitudinal ridges and strongly structured by reticulations; monospondylous trunk vertebrae centra 37–42, diplospondylous precaudal centra 37–45, total centra 125–140. Branchial, trunk and lateral caudal-fin dermal denticles loosely set, their surface very strongly and fully structured by reticulations. Claspers rather long and very slender, gradually narrowing to bluntly pointed tip without knob-like apex, inner margin length 10.1–11.3% TL, base width 1.4–1.5% TL; clasper hooks present along inner edge of large exorhipidion, large envelope overlapping part of clasper groove, inner lobe with rhipidion, cover rhipidion, pseudopera and pseudosiphon. The reproductive mode is yolk-sac viviparous. *Bythaelurus stewarti* n. sp. differs from all congeners in the distribution, which is apparently restricted to the Error Seamount. It further differs from all congeners in a higher spiral valve turn count (11–12 vs. 6–10) and in the morphology of branchial, trunk and lateral caudal-fin dermal denticles, which are loosely-spaced and not overlapping even in adult specimens of the new species, whereas they are closely-set and overlapping in all other *Bythaelurus* species. Compared to its morphologically and geographically closest congener, the new species further differs in a larger size, a ventral head with dark mottling, claspers that gradually narrow to the bluntly pointed tip without knob-like apex, and a surface of dermal denticles that is very strongly and fully structured by reticulations.

#### Description of the holotype

Values of the paratypes are presented in parentheses (n = 119 instead of n = 120 as the morphometrics of one paratype with deformed caudal fin were excluded). More complex differences between holotype and paratypes are described separately. Morphometric measurements and meristics are given in Table 1.

**Fig 1:**
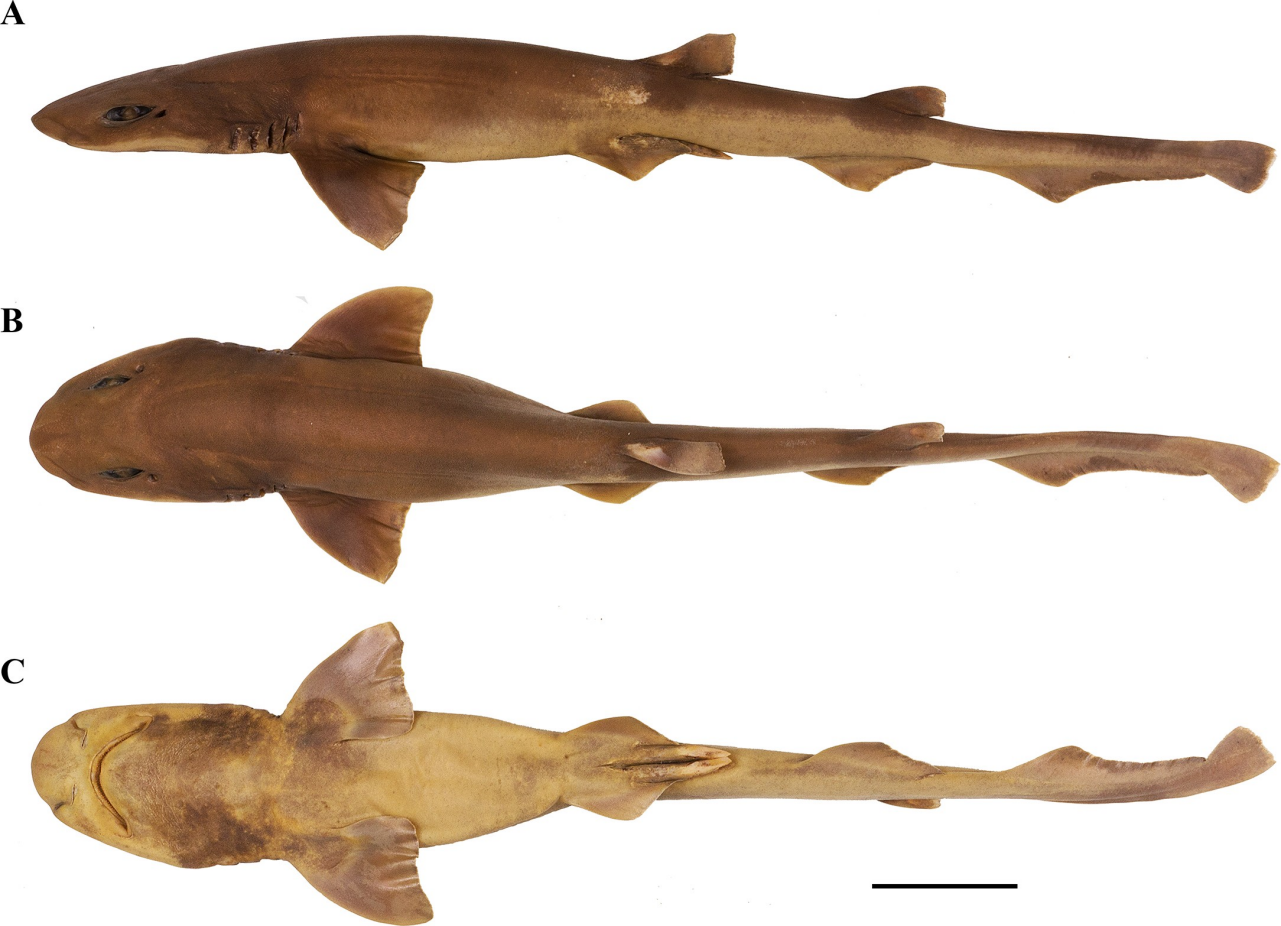
***Bythaelurus stewarti* n. sp., holotype, ZMH 26251, adult male, 425 mm TL, in (A) lateral, (B) dorsal, and (C) ventral views.** Scale bar: 5 cm.

**Fig 2:**
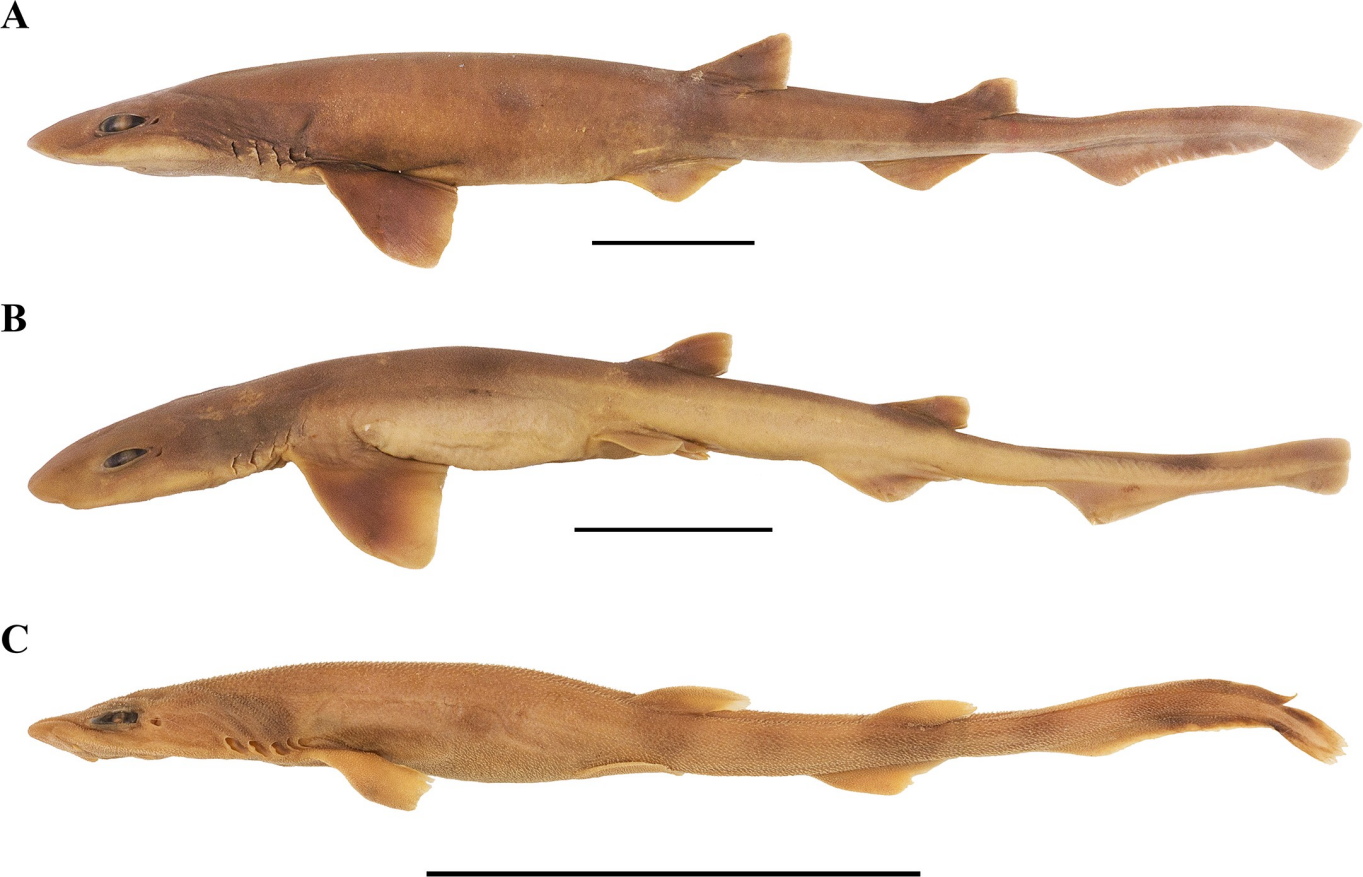
***Bythaelurus stewarti* n. sp., (A) paratype, ZMH 26253, gravid female, 425 mm TL, (B) paratype, ZMH 26252, juvenile male, 340 mm TL, and (C) paratype, ZMH 26253, female embryo, 137.3 mm TL in lateral views.** Scale bars: 5 cm.

**Fig 3:**
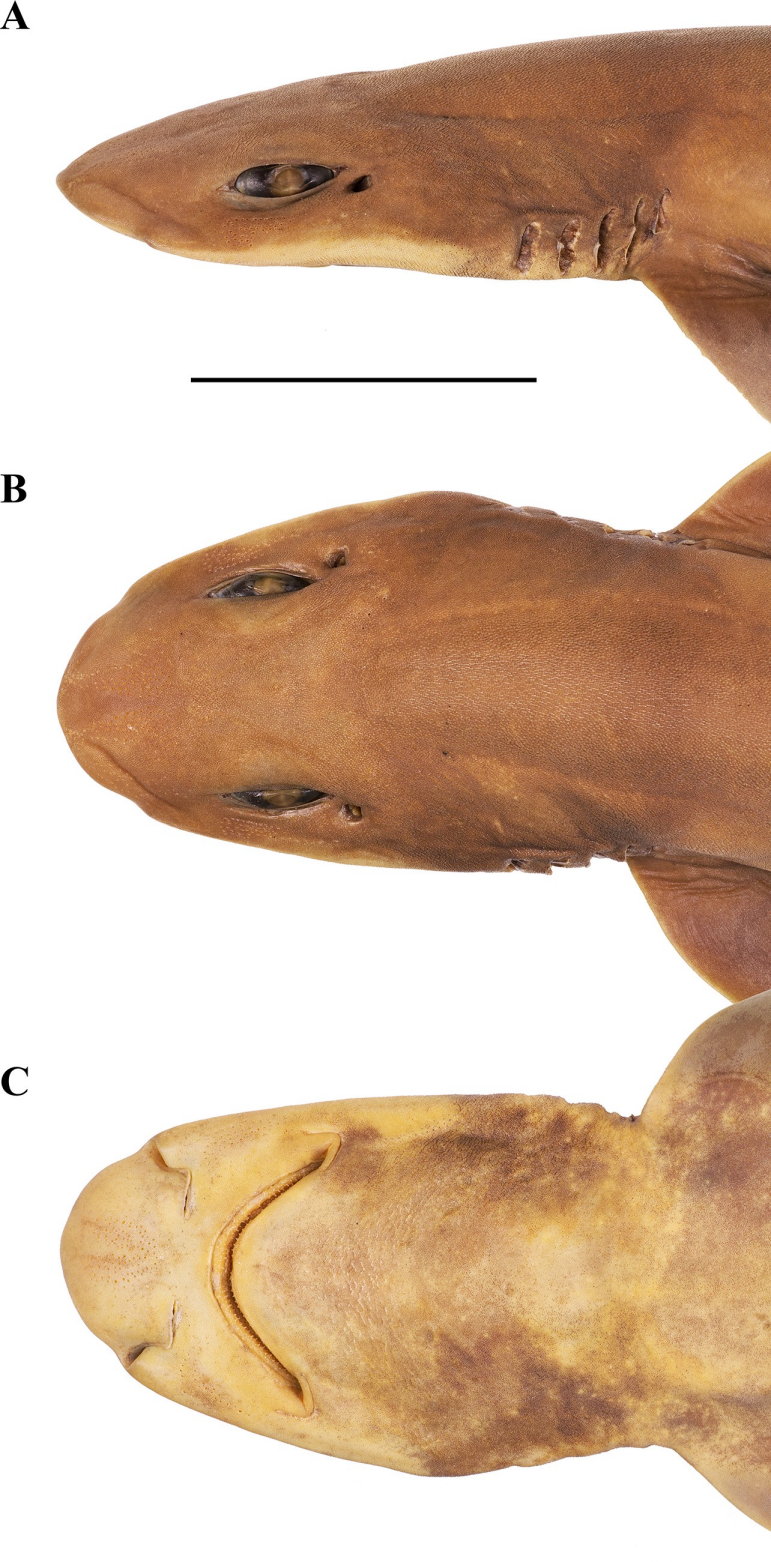
***Bythaelurus stewarti* n. sp., holotype, ZMH 26251, adult male, 425 mm TL, head in (A) lateral, (B) dorsal, and (C) ventral views.** Scale bar: 5 cm.

**Fig 4:**
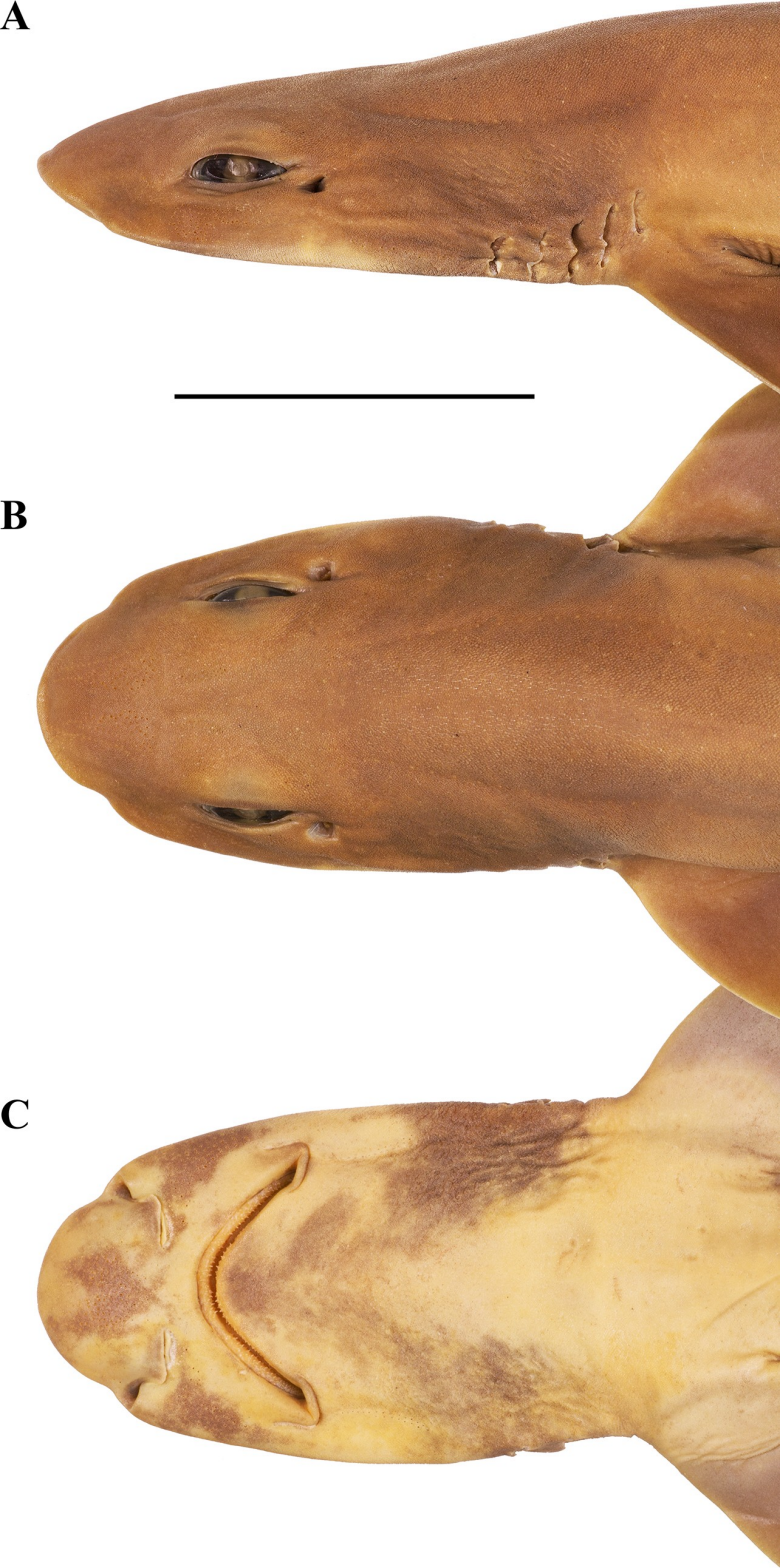
***Bythaelurus stewarti* n. sp., paratype, ZMH 26254, adult male, 417 mm TL, head in (A) lateral, (B) dorsal, and (C) ventral views.** Scale bar: 5 cm.

**Fig 5:**
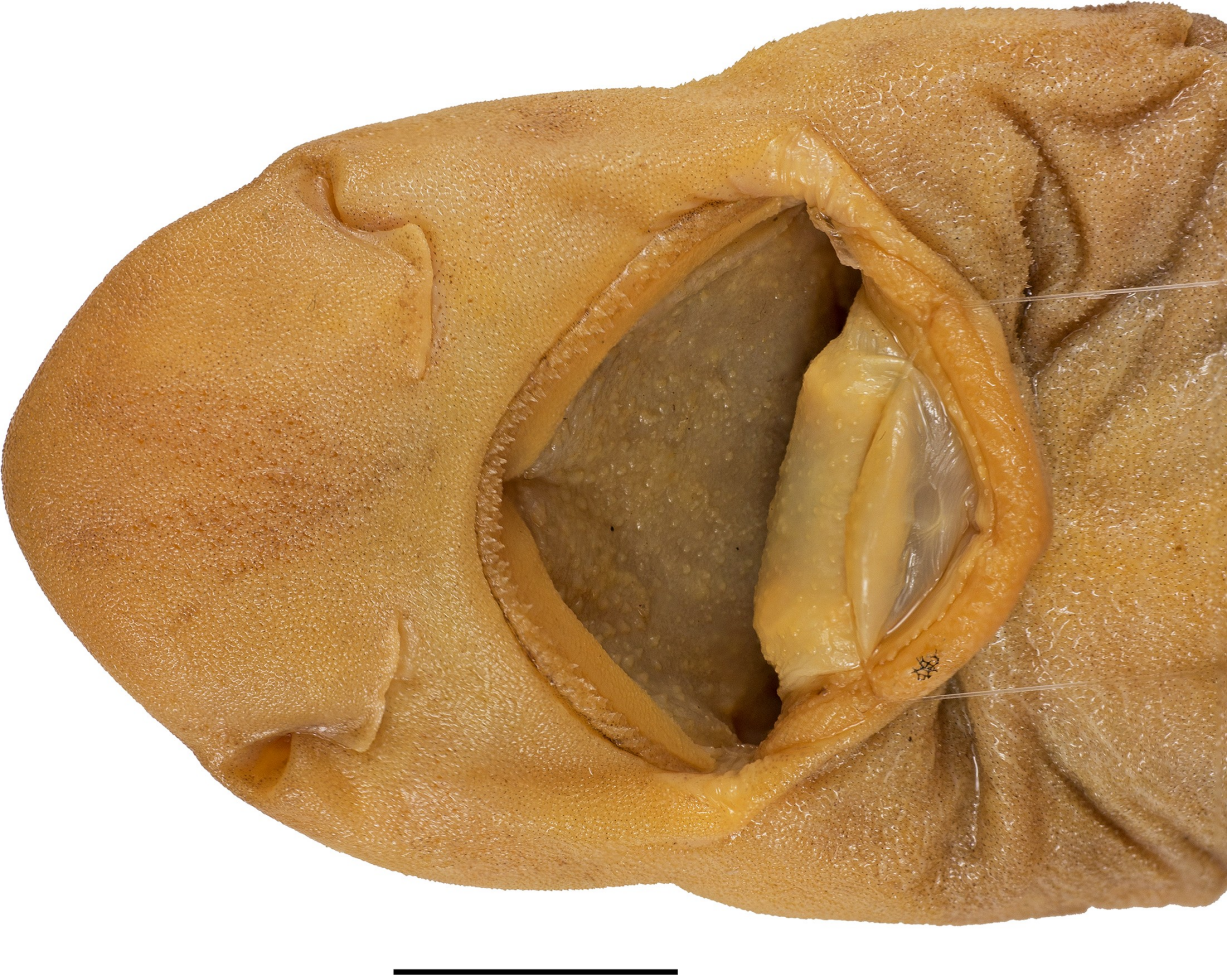
*Bythaelurus stewarti* n. sp., paratype, ZMH 26254, juvenile male, 266.5 mm TL, head in ventral view. Mouth spread open to show densely-set oral papillae on roof of mouth and tongue, as well as fleshy buccal curtain along inner margins of jaws. Scale bar: 1 cm. Focus-stacked image.

**Fig 6:**
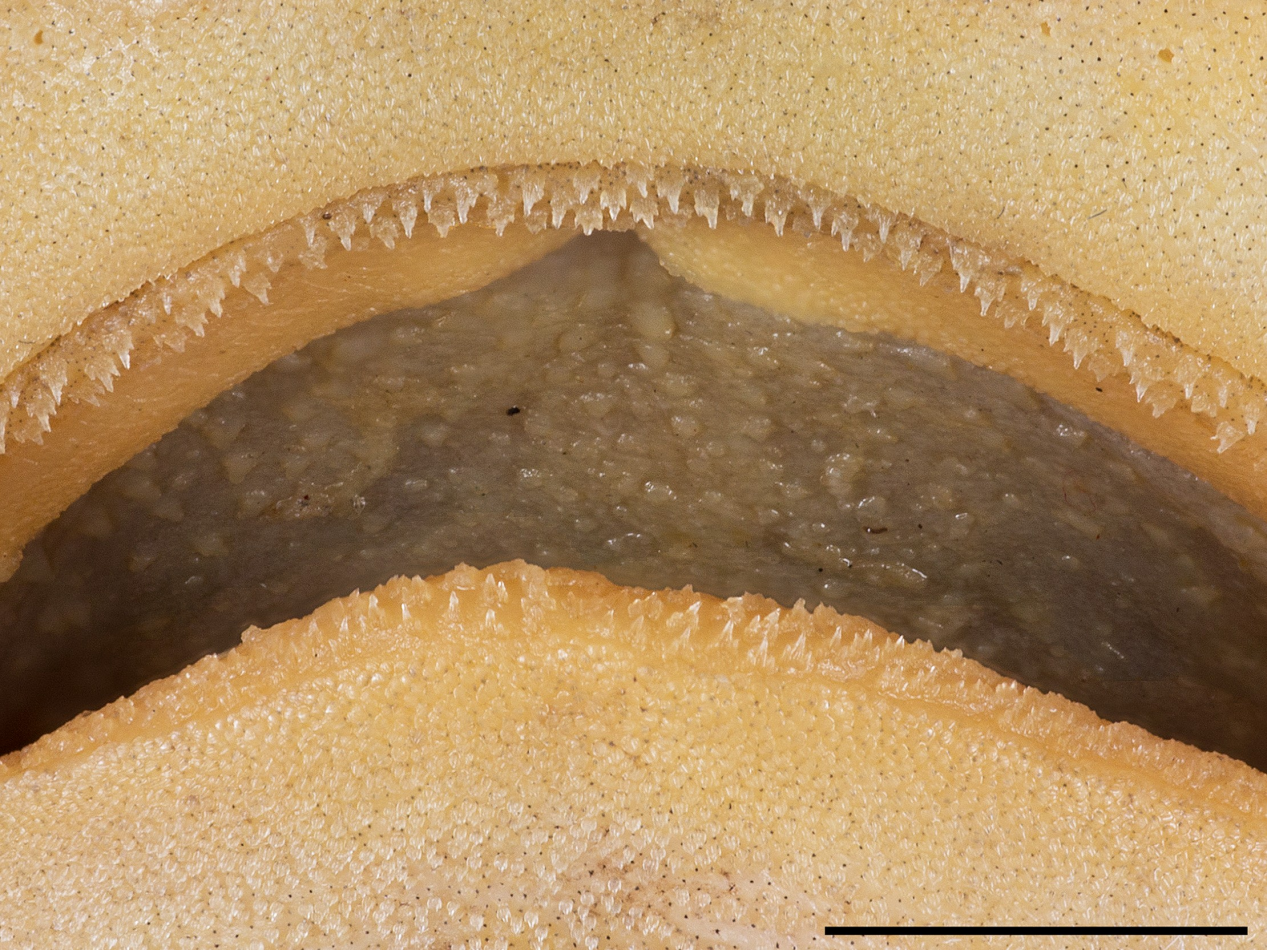
*Bythaelurus stewarti* n. sp., paratype, ZMH 26254, juvenile male, 266.5 mm TL, close-up of tooth rows, buccal curtains and oral papillae on roof of mouth. Scale bar: 0.5 cm. Focus-stacked image.

**External morphology**. Body firm and slender, subcircular in cross section at mid-trunk, laterally compressed and tapering posterior to cloaca; head region broad, long abdominal and caudal sections (Figs 1 and 2). No predorsal, interdorsal, or postdorsal ridges; no postanal ridge; no lateral ridges on caudal peduncle. Trunk shorter than tail, distance from tip of snout to anterior cloaca 45.1% TL (39.6–45.8% TL); pre-first dorsal-fin length 46.7% TL (41.8–47.6% TL), pre-second dorsal-fin length 64.4% TL (57.5–65.1% TL), ventral precaudal length 71.4% TL (66.1–71.8% TL). Head broad and dorsoventrally flattened, with a broadly rounded snout; laterally slightly compressed in gill region (Figs 3 and 4); no supraorbital crests on chondrocranium; head length 2.6 (2.2–2.6) times width at level of maximum extent of anterior nostrils and 1.3 (1.1–1.7) times pectoral—pelvic space; head width at level of maximum outer extent of anterior nostrils 1.2 (1.1–1.3) times width at level of lateral indention of head, 1.5 (1.2–1.6) times preorbital length, and 8.1% (8.2–10.1%) TL; head width at posterior edge of nostrils 1.4 (1.3–1.4) and at mouth corners 1.8 (1.4–1.8) times width at level of lateral indention of head; head width at middle gill slits 1.7 (1.0–1.7) times width at level of lateral indention of head. Snout long and broad, its tip broadly rounded, strongly bell-shaped in dorsoventral view with distinct lateral indention; pre-outer nostril length 1.0 (0.6–1.4) times internarial width and 0.5 (0.2–0.5) times interorbital width; preoral length 0.7 (0.6–1.2) times mouth width and 1.1 (0.8–1.2) times preorbital length; preorbital length 3.8 (2.8–4.5) times in head length and 0.9 (0.7–1.1) times interorbital space. Eyes moderately small and elongated, dorsolaterally on head, eye length 6.2 (4.9–7.7) times in head length, 13.7 (10.2–15.5) times in predorsal distance, and 2.1 (1.2–2.3) times eye height; nictitating lower eyelids, anterior and posterior eye notches, and suborbital grooves present. Spiracles small and slit-like, close behind but well separated from eyes, dorsolaterally on head and somewhat lower than level of eye notches, spiracle length 4.2 (2.9–16.8) times in eye length and 7.9 (5.9–32.1) times in interorbital width. Gill slits moderately long, well separated, their upper ends clearly below level of lower edge of eye; gill area fully scaled, gill filaments not visible externally; gill openings increasing in size from first to fourth (first to third or fourth), the fifth smallest and above pectoral-fin origin. Nostrils oblique, expanding diagonally inwards from snout edge, clearly not reaching level of mouth, with large, triangular anterior nasal flaps and much smaller but still distinct posterior flaps; pre-outer nostril length 1.0 (0.6–1.5) times nostril width and 0.5 (0.4–0.6) times preoral snout length, nostril width 1.0 (0.7–1.4) times internarial width and 0.8 (0.7–1.3) times eye length. Mouth broad, width 1.4 (0.8–1.7) times preoral length, 0.7 (0.6–0.7) times head width at mouth corners, 2.5 (2.4–4.8) times in head length, and 2.4 (1.5–3.2) times mouth length. Upper and lower labial furrows well developed, upper ones not reaching midpoint between mouth corner and posterior margin of nostril, lower furrows 1.2 (0.8–2.2) times as long as upper ones. Tongue moderately long, flat and rounded, light-colored, densely set with small, knob-like papillae; entire roof of mouth densely set with moderately small, knob-like papillae (Fig 5). Fleshy buccal curtain along inner margin of upper jaw extremely densely set with small, knob-like papillae, fleshy buccal curtain along inner margin of lower jaw loosely set with few very large, elongated, partially furcated papillae (Fig 6).

**Fig 7:**
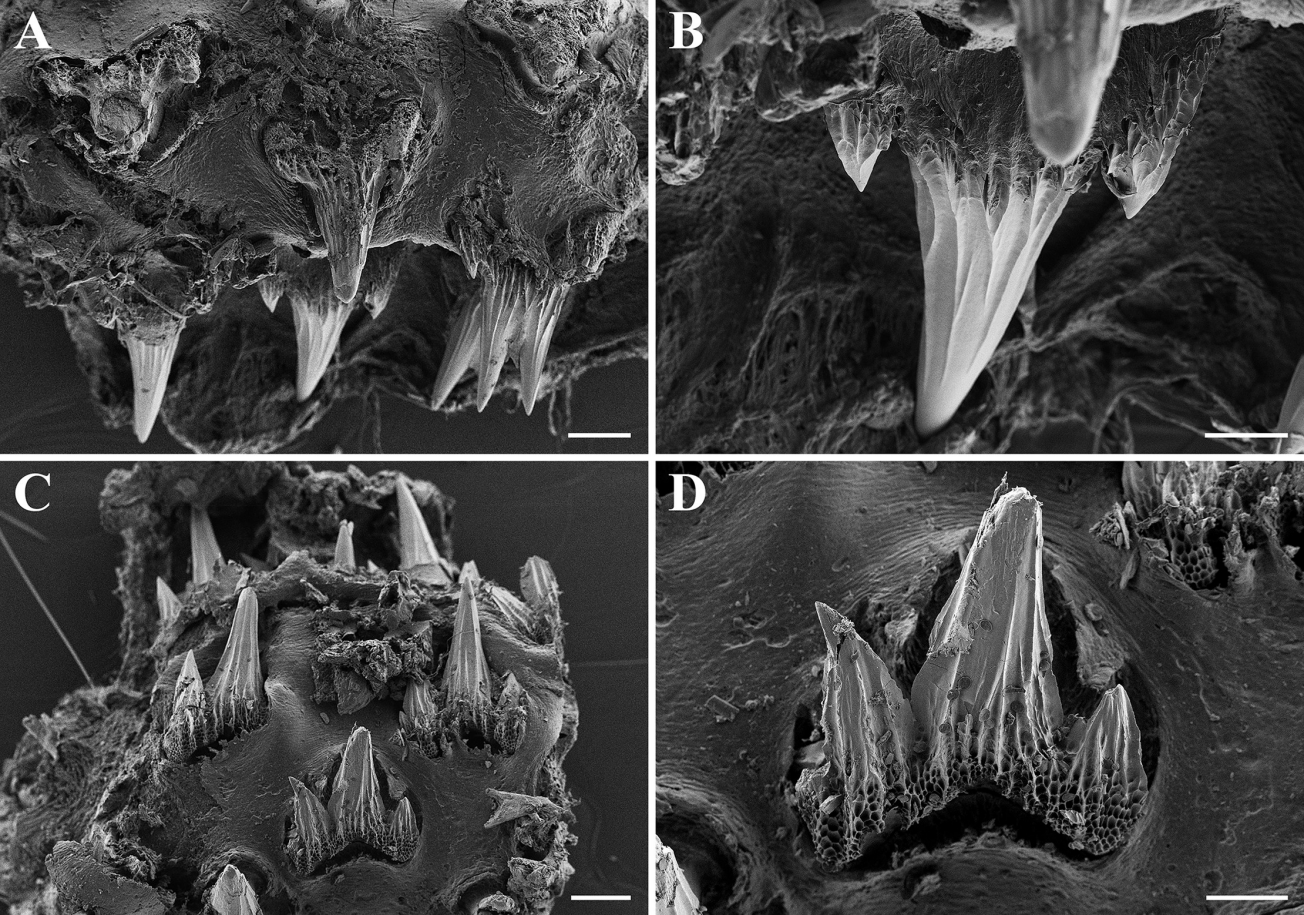
*Bythaelurus stewarti* n. sp., paratype, ZMH 26254, adult male, 375 mm TL, SEM images of tooth rows and single teeth. (A, B) Anterolateral teeth in upper jaw, (C, D) anterolateral teeth in lower jaw. Scale bars: (A, C) 200 μm, (B, D) 100 μm.

Upper jaw with approximately 77 (64–85) and lower jaw with about 74 (64–88) diagonal rows of small teeth (n = 50; Fig 6). Teeth in upper jaw tricuspidate with median cusp much longer than small lateral cusps (Fig 7A and 7B); teeth in lower jaw similar to those of upper jaw but with larger lateral cusps (Fig 7C and 7D). The median cusp decreases in size from symphysis to mouth corners, whereas the size of the lateral cusps increases, reducing the size difference of median and lateral cusps towards mouth corners in both jaws. Outer surface of crown furrowed by strong longitudinal ridges from base of cusps to tip and strongly structured by reticulations from basal areas to slightly into median cusp but to well into lateral cusps. Cutting edges of cusps without serrations.

**Fig 8:**
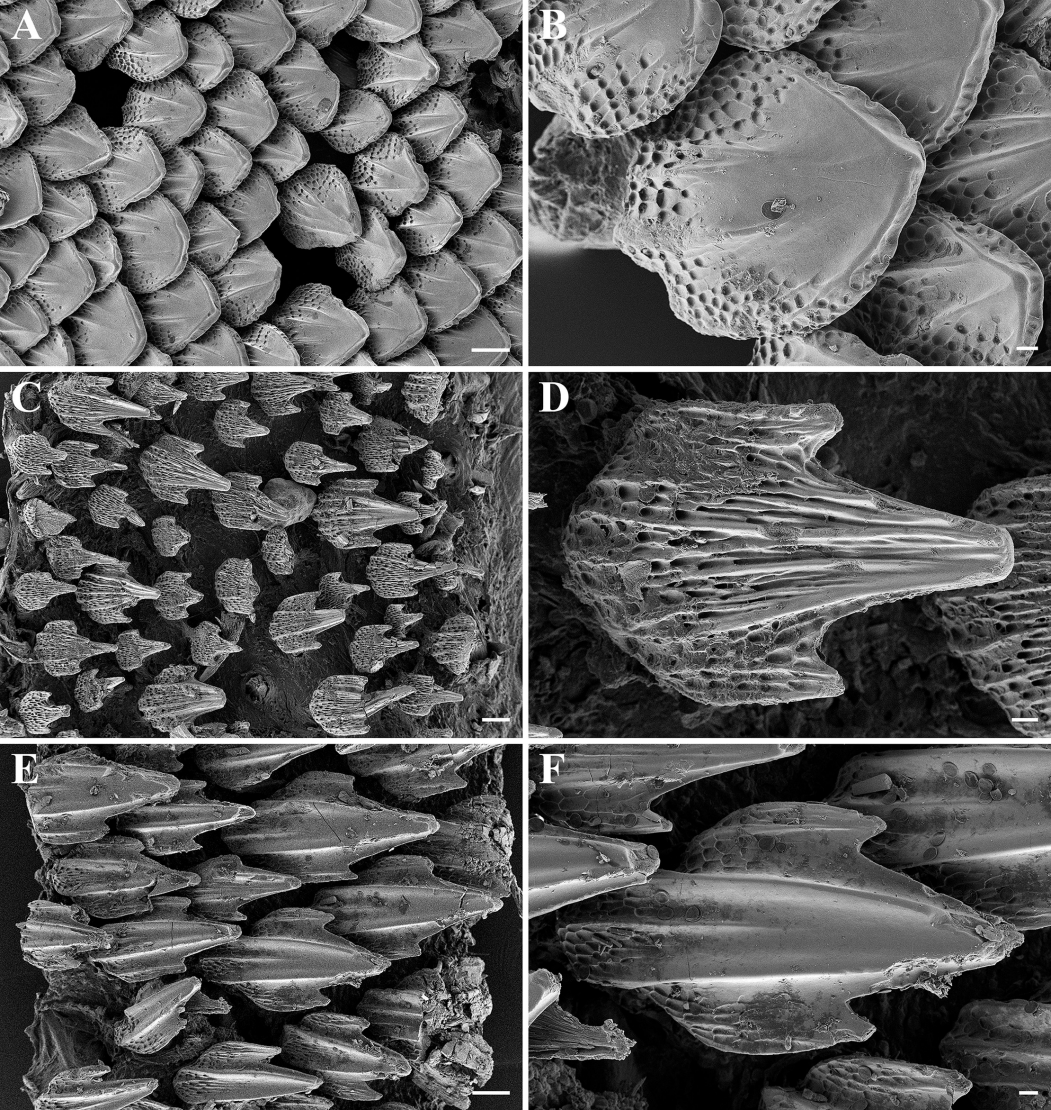
*Bythaelurus stewarti* n. sp., paratype, ZMH 26254, adult male, 375 mm TL, SEM images. Dermal denticles on (A–B) ventral snout tip, (C–D) lateral trunk, and (E–F) anterior dorsal caudal-fin margin. Scale bars: (A, C, E) 100 μm, (B, D, F) 20 μm.

**Fig 9:**
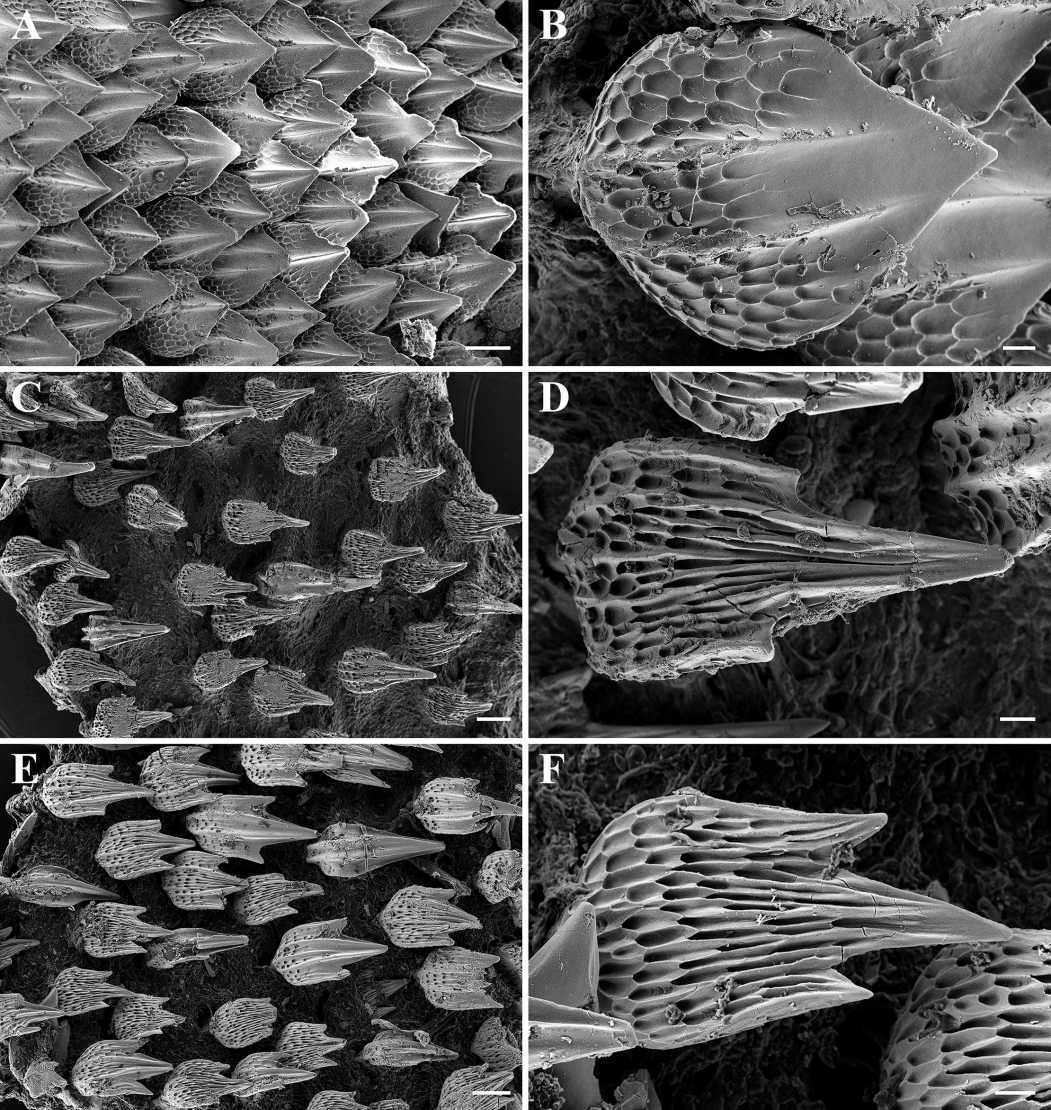
*Bythaelurus stewarti* n. sp., paratype, ZMH 26252, juvenile male, 291 mm TL, SEM images. Dermal denticles on (A–B) ventral snout tip, (C–D) lateral trunk, and (E–F) anterior dorsal caudal-fin margin. Scale bars: (A, C, E) 100 vm, (B, D, F) 20 μm.

**Fig 10:**
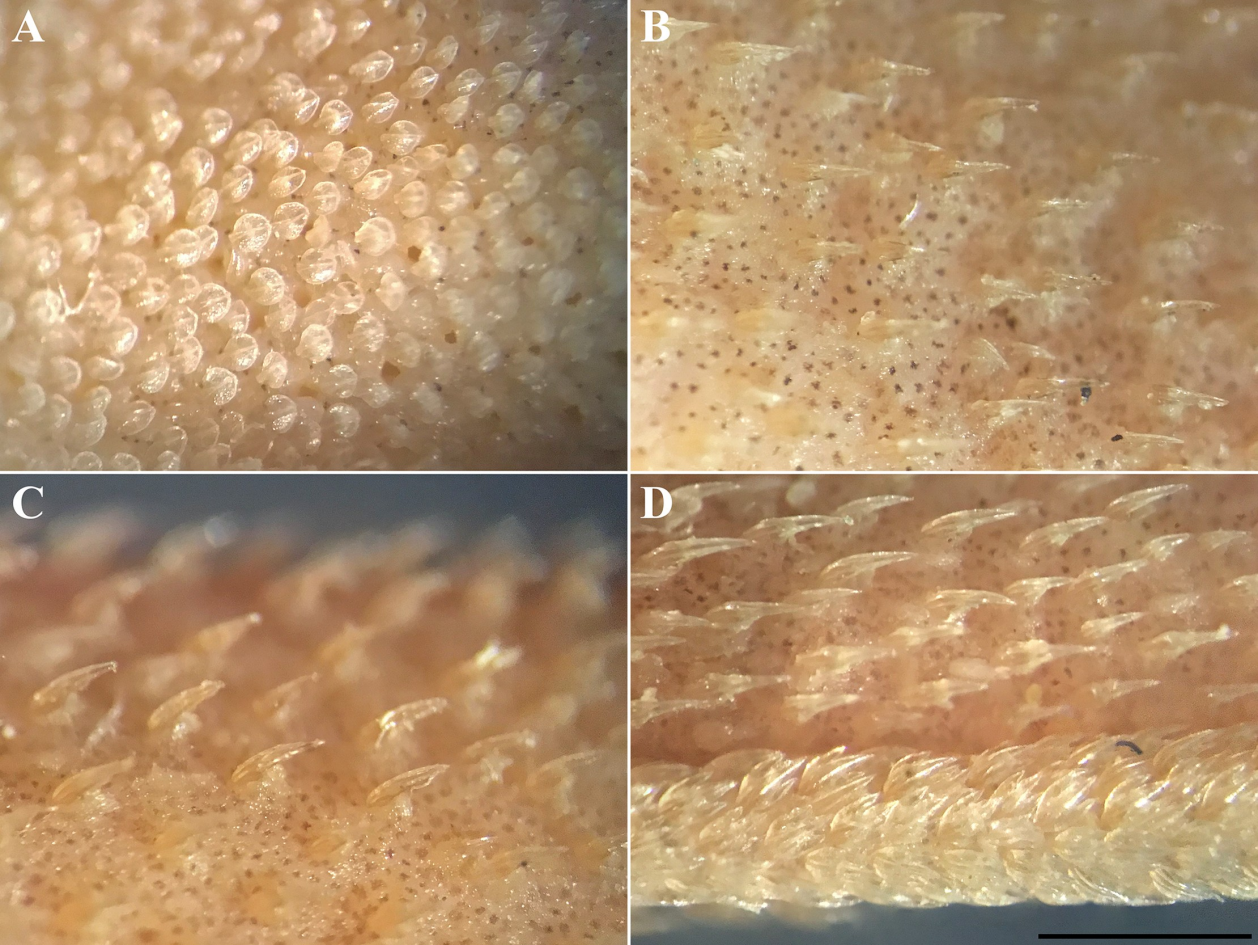
*Bythaelurus stewarti* n. sp., paratype, ZMH 26253, female embryo, 137.3 mm TL, microscopic images. Dermal denticles on (A) ventral snout tip, (B) lateral trunk in lateral view, (C) lateral trunk in dorsal view, and (D) anterior caudal fin depicting crest of slightly enlarged denticles on dorsal margin, as well as lateral caudal-fin denticles (focus-stacked image). Scale bar: 1 mm.

Dermal denticles on dorsal and ventral (Fig 8A and 8B) snout leaf-like to teardrop-shaped, densely set and overlapping, surface structured by reticulations in basal third, with two to four narrow ridges that do neither fuse nor reach the tip of the denticle. Snout denticles in juveniles of similar shape and also densely set and overlapping, but surface structured by reticulations in basal half and with only two to rarely three ridges that partially fuse and partially reach the tip of the denticle (Fig 9A and 9B). Denticles in branchial area, on lateral trunk (Fig 8C and 8D) and on lateral caudal fin tricuspidate, small, loosely set and not overlapping, with long and pointed median main cusp and shorter, pointed lateral cusps at lower level, surface very strongly and fully structured by reticulations, with about four narrow ridges that partially reach the tip of the median main cusp but are hardly detectable due to being camouflaged by the strong and dense reticulations. Branchial, trunk (Fig 9C and 9D) and lateral caudal-fin denticles in juveniles similar but even less densely set and partially unicuspidate without lateral cusps. Dermal denticles on anterior dorsal caudal-fin margin (Fig 8E and 8F) slightly enlarged, tricuspidate, densely set and overlapping, with long and pointed median main cusp and shorter, pointed lateral cusps at lower level, surface only weakly structured by reticulations only close to base, with a weak median ridge in basal half and two very strong and pronounced lateral ridges that do not fuse but reach the tip of the median main cusp, two additional, very weak lateral ridges in lateral cusps that reach or nearly reach the tip of the lateral cusps. Dermal denticles on anterior dorsal caudal-fin margin in juveniles (Fig 9E and 9F) strongly differ from those of adults, not enlarged, tricuspidate, loosely set and not overlapping, with long and pointed median main cusp and shorter, pointed lateral cusps at lower level (sometimes absent), surface very strongly and fully or mostly structured by reticulations, partially with a median ridge in basal half and two lateral ridges that fuse and reach the tip of the median main cusp, but ridges not present or hardly detectable due to being camouflaged by the strong and dense reticulations in about one third to half of the denticles. In embryos, the denticles are similar in shape on the snout but less densely set and hardly overlapping (Fig 10A), unicuspidate, as well as very loosely set and not overlapping in branchial area, on lateral trunk (Fig 10B and 10C), and on lateral caudal fin (Fig 10D), but tricuspidate on anterior dorsal caudal-fin margin (Fig 10D). In contrast to larger specimens, the latter denticles are somewhat smaller in size than those on the lateral caudal fin but still forming an obvious crest (Fig 10D).

Pectoral fins subtriangular, non-falcate, anterior margin weakly convex, the length 2.3 (1.6–3.0) times pectoral base length and 1.5 (1.1–2.5) times length of the slightly concave posterior margin, apex narrowly rounded, inner margin convex and 1.0 (0.7–2.6) times pectoral base length, inner pectoral corner broadly rounded (Figs 1 and 2). Pectoral—pelvic space 1.3 (1.1–1.9) times length of pectoral-fin anterior margin and 1.4 (1.2–2.1) times interdorsal space.

**Fig 11:**
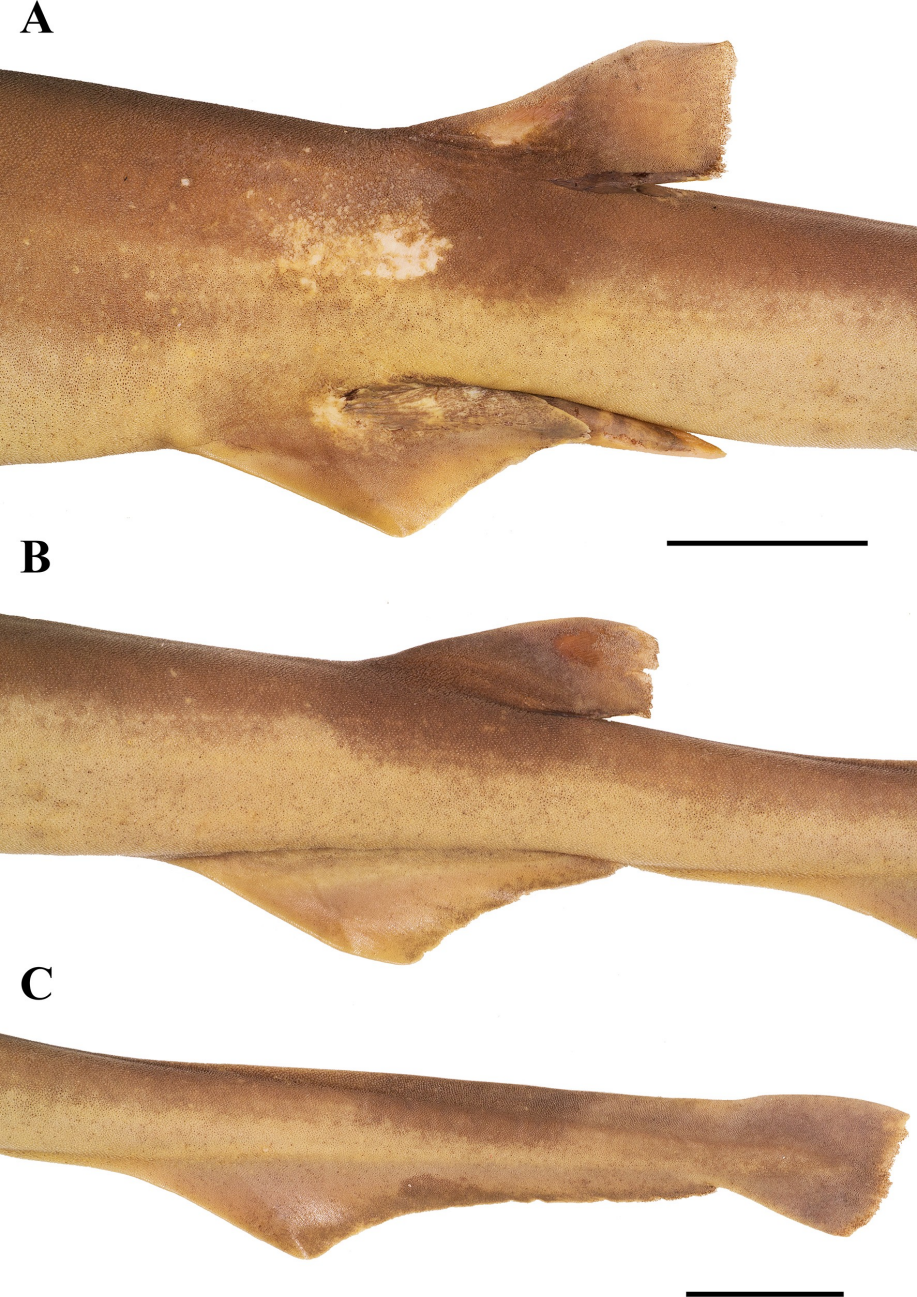
*Bythaelurus stewarti* n. sp., holotype, ZMH 26251, adult male, 425 mm TL, lateral views of fins. (A) First dorsal and pelvic fins, (B) second dorsal and anal fins, and (C) caudal fin. Scale bars: 2 cm. Note ventral precaudal ridge in (B) and (C).

Pelvic fins narrowly triangular with long, straight anterior margin, slightly concave posterior margin and short, straight inner margin, anterior margin 0.4 (0.3–0.6) times pectoral-fin anterior margin, apex narrowly rounded; pelvic-fin origin clearly anterior to first dorsal-fin origin, pelvic posterior tips about level with insertion of first dorsal fin (Fig 11A). Pelvic—anal space relatively long, 12.6% (8.0–12.7%) TL and 0.8 (0.5–0.8) times pectoral—pelvic space.

First dorsal fin 1.4 (0.9–2.1) times as high and 1.1 (0.9–1.3) times as long as second dorsal fin, anterior margin straight (straight to slightly convex), apex angular, posterior and inner margins straight, free rear tip angular; base length 1.5 (1.1–2.5) times fin height and 0.5 (0.4–0.8) times interdorsal space; first dorsal-fin origin over pelvic-fin midbase (Fig 11A).

Second dorsal fin 0.7 (0.5–1.1) times as high as and about as long as first dorsal fin, anterior margin slightly convex, apex narrowly rounded, posterior and inner margins straight, free rear tip angular, base length 2.2 (1.6–4.0; no ontogenetic or sexual differences detectable) times fin height and 0.5 (0.4–1.1) times interdorsal space; second dorsal-fin origin over anal-fin midbase (Fig 11B).

Anal fin a long and relatively low triangle, with long, slightly convex anterior margin, slightly shorter and slightly concave posterior margin, and short inner margin, apex angular, free rear tip pointed angular; base length 3.3 (2.6–6.0) times fin height, 0.8 (0.7–1.9) times interdorsal space, and 0.8 (0.8–1.6) times pelvic—anal space; base 1.7 (1.4–2.3) times longer than second dorsal-fin base and 5.8 (6.1–32.7; large variation due to partially very small anal—caudal space, no ontogenetic or sexual differences detectable) times anal—caudal space (Fig 11B). Anal-fin origin distinctly anterior to second dorsal-fin origin.

Caudal fin slender, relatively long (28.2–34.4% TL) and strongly asymmetrical, its length 5.0 (4.1–5.8) times fin height and 2.3 (2.3–3.7) times interdorsal space; dorsal caudal margin weakly convex, no lateral undulations; upper caudal lobe very low, lower caudal lobe much deeper, with straight pre- and postventral margins. Ventral caudal-fin origin anterior of dorsal caudal-fin origin due to long preventral margin, which is slightly shorter than the postventral margin and forms an elongated fleshy ridge in about anterior half of its length. Ventral corner bluntly angled; subterminal notch distinct; terminal lobe 5.6 (4.1–9.2) times in caudal-fin length; terminal caudal margin nearly straight without mesial indention (Fig 11C).

**Fig 12:**
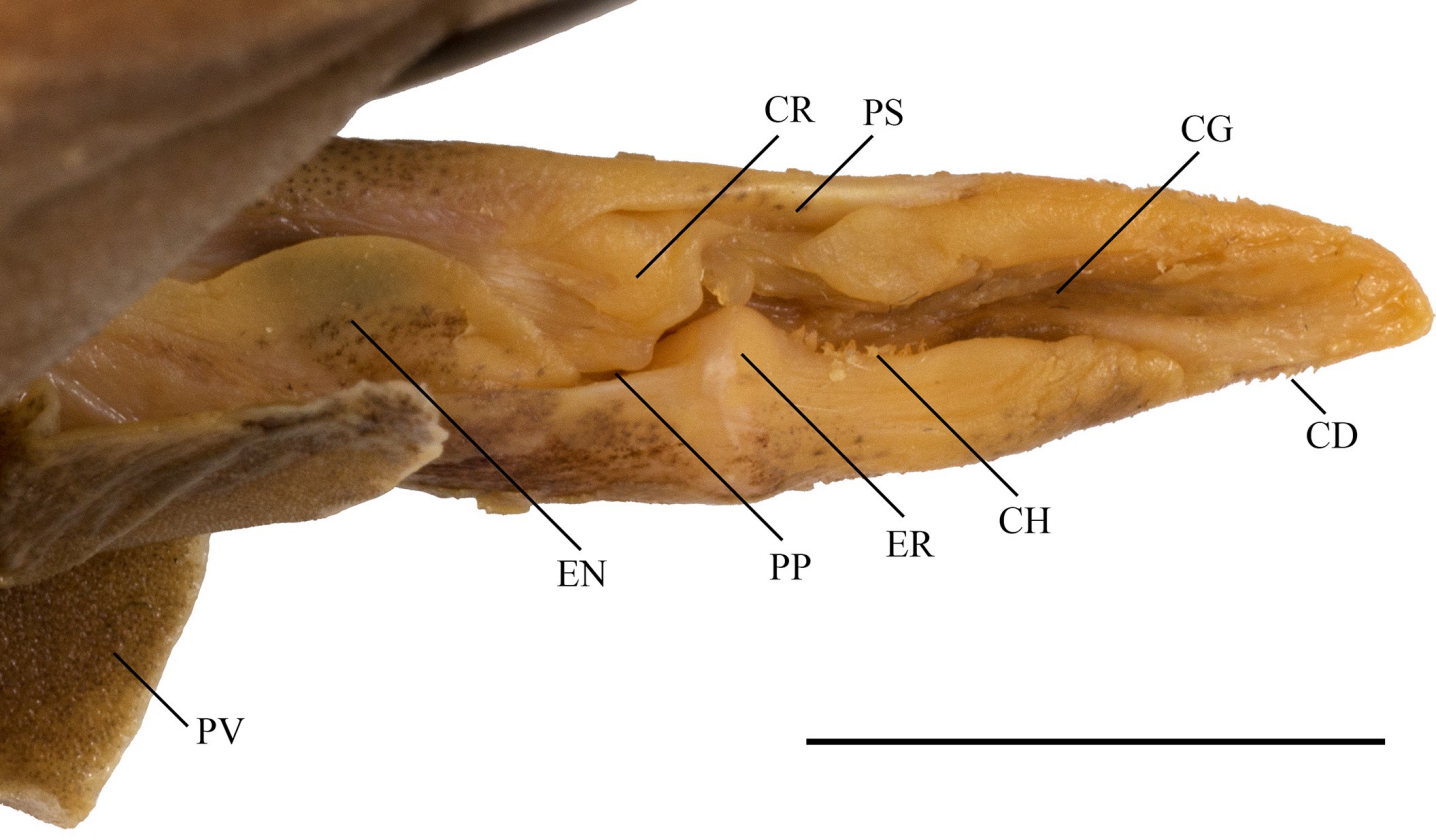
*Bythaelurus stewarti* n. sp., paratype, ZMH 26254, adult male, 391 mm TL, left clasper in dorsal view. CD = clasper denticles; CG = clasper groove; CH = clasper hooks; CR = cover rhipidion; EN = envelope; ER = exorhipidion; PP = pseudopera; PS = pseudosiphon; PV = pelvic fin. Scale bar: 1 cm.

Claspers (Fig 12) of mature males rather long, terminating distinctly before anal-fin origin and very slender, lateral margins nearly straight, not undulated, extending to about one third of their inner margin length beyond pelvic-fin free rear tips; inner margin length 10.1% (10.4–11.3%) TL, base width 1.4% (1.4–1.5%) TL. Glans somewhat elongated, length about half clasper inner margin; only slightly tapering to tip in distal half and gradually narrowing to bluntly pointed tip without knob-like apex. Ventral and outer lateral surfaces of clasper covered with small tricuspidate clasper denticles (CD), similar to those on trunk; dorsal and inner lateral surfaces largely naked. The narrow slit-like apopyle opens the clasper groove proximally; the hypopyle ends the concealed clasper groove distally and is detectable as a small cavity next to the rhipidion, but both are concealed by the cover rhipidion and exorhipidion and thus not visible in Fig 12. The proximally concealed clasper groove (CG) opens widely in the distal glans. A large, fleshy flap, the envelope (EN), on outer lobe of glans, overlaps part of CG; outer lobe also with a large, subtriangular exorhipidion (ER), which consists of a proximal convex blade and a distal fleshy wall; several enlarged clasper denticles, the clasper hooks (CH) along inner edge of ER. Inner lobe with a fan-shaped flap, the rhipidion, that partially covers the concealed part of CG and itself is concealed by a movable blade, the large cover rhipidion (CR); inner lobe also with two blind cavities: the large and deep pseudopera (PP), which is partially concealed by EN and RH, and–on the inner margin–the large, longitudinally slit-like pseudosiphon (PS).

Skeletal meristics (from radiographs, n = 121, Table 1): monospondylous trunk vertebral centra: 38 (37–42); diplospondylous precaudal centra: 43 (37–45); total precaudal centra: 81 (75–84); caudal centra: 55 (44–59); total centra: 136 (125–140). Spiral valve turns (n = 2): 11–12.

**Coloration**. Fresh, prior to preservation: dorsally dark grayish-brown, dark blotches at nape, on flank between pectoral and pelvic fin, dorsolaterally on body below first and second dorsal fins, and one across caudal fin; all blotches rather indistinct due to dark ground coloration; ventral side grayish-white with dark grayish-brown mottling on head. Color in preservative: dorsally medium-dark brown, dark brown blotches somewhat more distinct than in fresh condition likely due to slight fading of the ground coloration; ventral side beige with dark brown mottling on head, particularly distinct in larger specimens. Fins similar in color to body, but gradually brightening towards their margins. Jaws, gums, and mouth cavity beige.

#### Size

**Fig 13:**
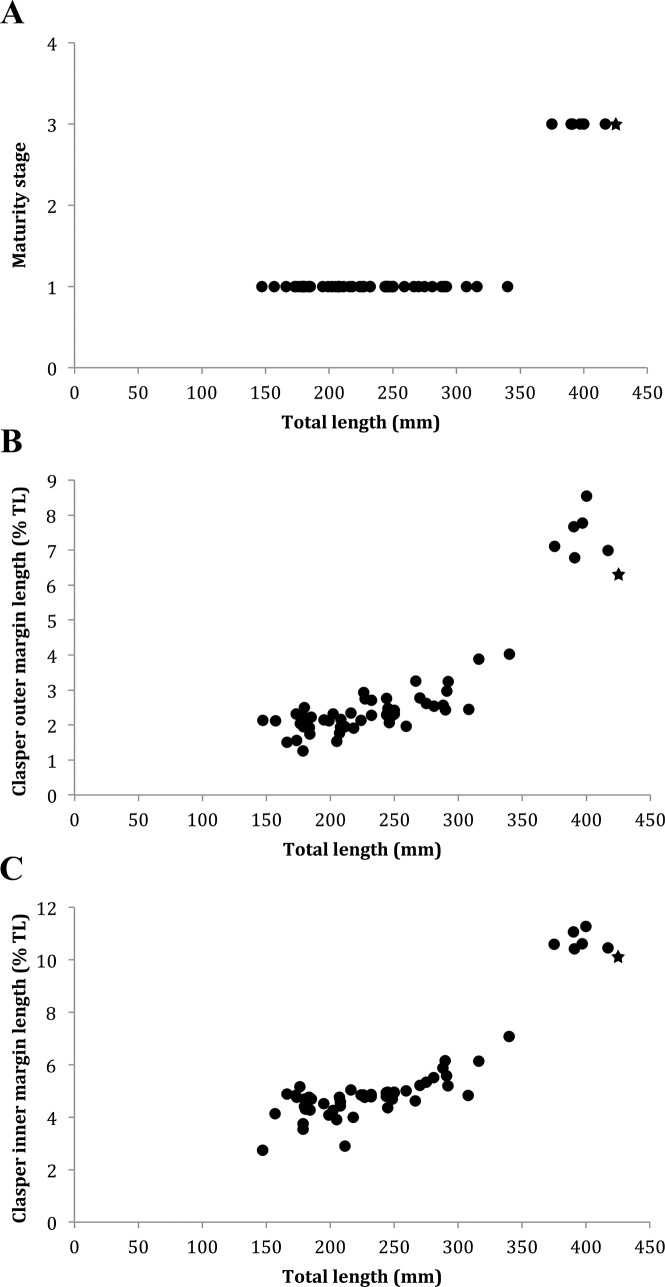
***Bythaelurus stewarti* n. sp.**, (A) maturity stages, (B) clasper outer margin length, and (C) clasper inner margin length of all male type specimens (black star = holotype, black circles = male paratypes).

The description of the size is based on total length measurements prior to preservation except for the two female near-term embryos measured in 70% ethanol. A small catshark and a medium-sized species of *Bythaelurus* reaching a maximum total length of about 437 mm for females and 435 mm for males. Males are mature at 389 mm and juvenile at 348 mm TL. The smallest known free-swimming specimens have total lengths of 156 (female) and 162 mm (male), respectively. The size at birth is estimated at around 150 mm based on the smallest free-swimming specimens and two female near-term embryos of 137.3 to 145.3 mm TL found in the gravid female. The maturity stages, as well as clasper outer and inner margin lengths of all male type specimens of the new species are shown in Fig 13.

#### Distribution

The new species is known only from the Error Seamount (Mount Error Guyot) in 380–420 m depth (see map in the Discussion section). It is apparently a microendemic species restricted to this isolated Seamount.

#### Etymology

The new species is named after the late filmmaker and shark conservationist Rob Stewart, who inspired the second author and stimulated her interest in sharks.

#### Discriminant function analyses for *Bythaelurus stewarti* n. sp. and *B*. *hispidus*

**Fig 14:**
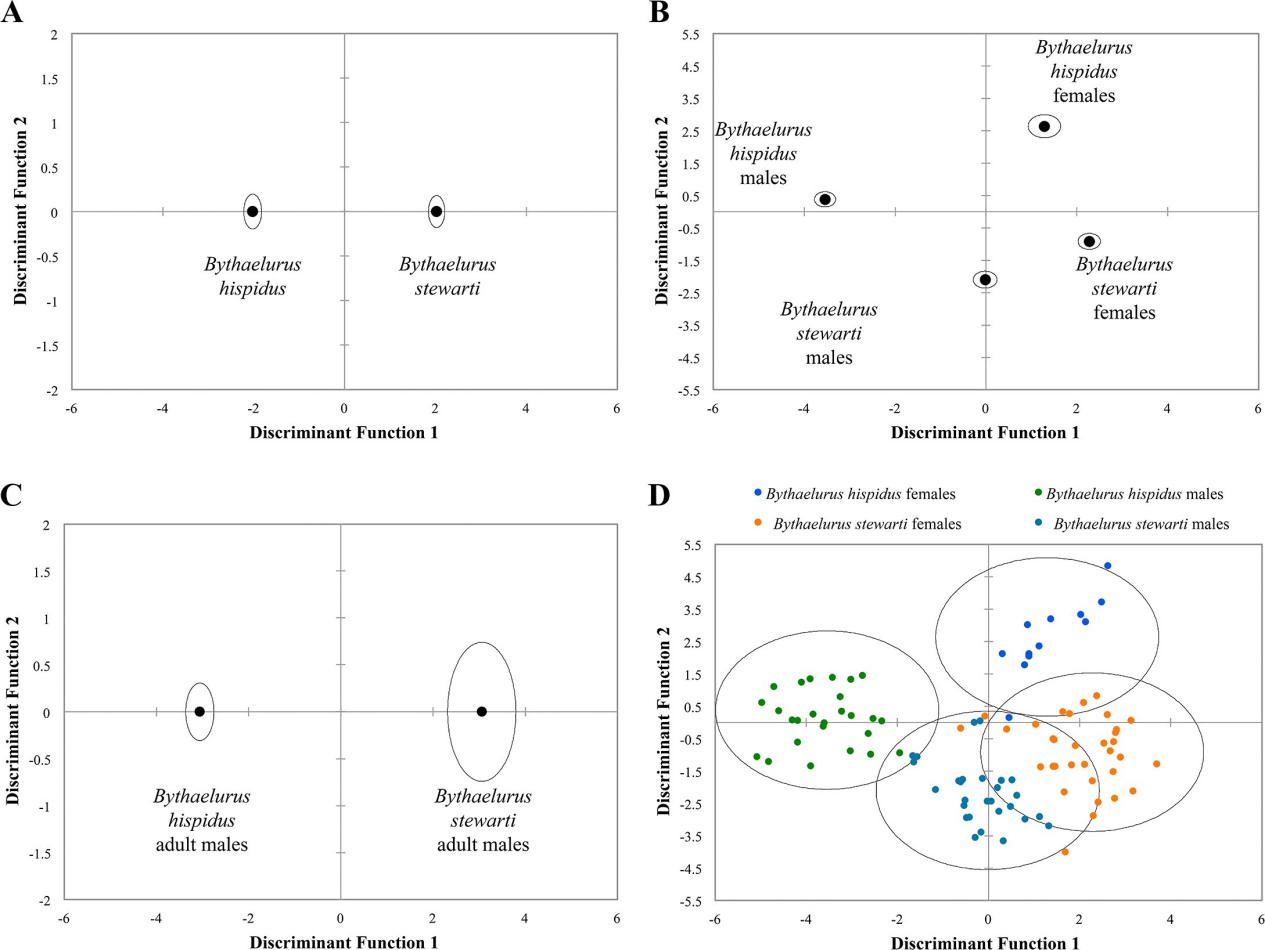
Plots of group centroids of *Bythaelurus stewarti* n. sp. and *B*. *hispidus*. (A) all analyzed specimens of *B*. *stewarti* n. sp. and *B*. *hispidus*, (B) males and females of *B*. *stewarti* n. sp. and *B*. *hispidus*, (C) adult males of *B*. *stewarti* n. sp. and *B*. *hispidus*. Circles represent 95% confidence intervals for group centroids. (D) Plot of all analyzed male and female specimens of *B*. *stewarti* n. sp. and *B*. *hispidus*. Circles include 95% of specimens in each group.

The first DFA for *Bythaelurus stewarti* n. sp. and *B*. *hispidus* provided one significant function (Box-Test with χ2 = 430.430 and p < 0.0001; Wilks’ lambda = 0.195 and p < 0.0001), which explains 100% of the total variation in the data. Group centroids for morphological characters of both species are projected in Fig 14A. Both species are clearly separated in the discriminant space defined by the first function. From the standardized coefficients (Table 2), the two characters that have the greatest influence on the discriminant function (characters most discriminatory) are the preoral length (POR) and caudal-fin postventral margin (CPoV).

**Table 2:** Standardized coefficients of the first discriminant function (DF1) of the first discriminant function analysis (DFA) separating the two species *Bythaelurus stewarti* n. sp. and *B*. *hispidus* based on morphological characters. In bold, characters with the greatest weight in DF1. For explanation of morphological characters, see Table 1.

Morphological characters	DF1
HDL_2	-0.204
ACS	-0.365
PCA	0.372
PONL	-0.308
POR	**0.511**
EYL	-0.286
ING	0.168
GS2	-0.219
GS3	0.198
P1I	-0.353
P1P	-0.349
D1L	0.222
D2A	0.295
D2I	-0.207
P2A	0.308
ANA	-0.234
CH	-0.241
CPrV	-0.186
CPoV	**0.455**
CTH	0.264
TAH	0.332
CPH	0.208
MOW	-0.432
Percentage of explained variance	100.000
Eigenvalue	4.132
Cumulative variance in %	100.000

In the second DFA, three significant functions were estimated based on morphological characters of males and females of *Bythaelurus stewarti* n. sp. and *B*. *hispidus* (Box-Test with χ2 = 944.979 and p < 0.0001; Wilks’ lambda = 0.025 and p < 0.0001). Together, these functions explain 100% of the total variation in the data. The first two functions explain 92.875% of the total variation in the data (Table 3), which is sufficient for further detailed analysis. The third discriminant function explains 7.125% of total variation. Group centroids for morphological characters of males and females of both species are projected in Fig 14B. Fig 14D presents the individual specimens projected onto the first two discriminant functions. As all four groups were clearly separated in the discriminant space defined by the first two functions, the third function was not used. The first discriminant function explains 56.666% of total variation (Table 3). It mainly separates the males of *Bythaelurus stewarti* n. sp. and *B*. *hispidus*. The females of these species cannot be clearly separated by the first discriminant function. The second discriminant function accounts for 36.210% of total variation. The females of *Bythaelurus stewarti* n. sp. and *B*. *hispidus* are clearly discriminated by this function. Pelvic height (P2H) and tail height at pelvic base end (TAH) are the two characters that have the greatest weight on the first discriminant function and for the discrimination of the males of both species (Table 3). The contrasts between the pelvic fin height (P2H) and the pelvic anterior margin length (P2A) are mainly responsible for the discrimination of the second discriminant function and for the discrimination of the females of both species (Table 3).

**Table 3:** Standardized coefficients of the first three discriminant functions (DF1, DF2, DF3) of the second discriminant function analysis (DFA) separating males and females of *Bythaelurus stewarti* n. sp. and *B*. *hispidus* based on morphological characters. In bold, characters with the greatest weight in DF1 and DF2. For explanation of morphological characters, see Table 1.

Morphological characters	DF1	DF2	DF3	
PRVC	-0.161	0.306		-0.163
PG1	-0.382	-0.061		0.183
PP1	-0.091	-0.002		0.337
PCA	0.358	-0.087		-0.337
POR	0.337	-0.134		0.166
P1I	-0.197	0.307		0.398
D1P	-0.129	0.002		-0.692
D2A	0.252	-0.146		-0.300
P2A	0.027	**-0.451**		-0.129
P2B	0.154	0.170		0.416
P2H	**0.873**	**0.471**		0.343
P2I	-0.316	0.064		-0.138
ANH	0.002	0.327		0.125
CPoV	0.335	-0.307		-0.022
CTH	-0.134	-0.237		0.310
TAH	**0.550**	0.074		-0.473
MOL	0.065	0.287		0.214
MOW	-0.141	0.364		-0.116
LLA	-0.093	-0.139		-0.346
ESL	0.096	0.175		-0.323
TAW	-0.157	-0.301		-0.007
trunk vertebral centra	0.071	-0.202		0.235
Percentage of explained variance	56.666	36.210		7.125
Eigenvalue	4.930	3.150		0.620
Cumulative variance in %	56.666	92.875		100.000

The third DFA for adult males of *Bythaelurus stewarti* n. sp. and *B*. *hispidus* provided one significant function (Box-Test with χ2 = 1153.665 and p < 0.0001; Wilks’ lambda = 0.093 and p < 0.0001), which explains 100% of the total variation in the data. Group centroids for morphological characters of adult males of both species are projected in Fig 14C. Adult males of both species are clearly separated in the discriminant space defined by the first function. From the standardized coefficients (Table 4), the two characters that have the greatest influence on the discriminant function are the total length (TL) and clasper base width (CLB).

**Table 4:** Standardized coefficients of the first discriminant function (DF1) of the third discriminant function analysis (DFA) separating the adult males of *Bythaelurus stewarti* n. sp. and *B*. *hispidus* based on morphometric characters. In bold, characters with the greatest weight in DF1. For explanation of morphological characters, see Table 1.

Morphological characters	DF1
TL	**1.024**
CLI	0.286
CLB	**-0.303**
Percentage of explained variance	100.000
Eigenvalue	9.761
Cumulative variance in %	100.000

### Feeding ecology

**Fig 15:**
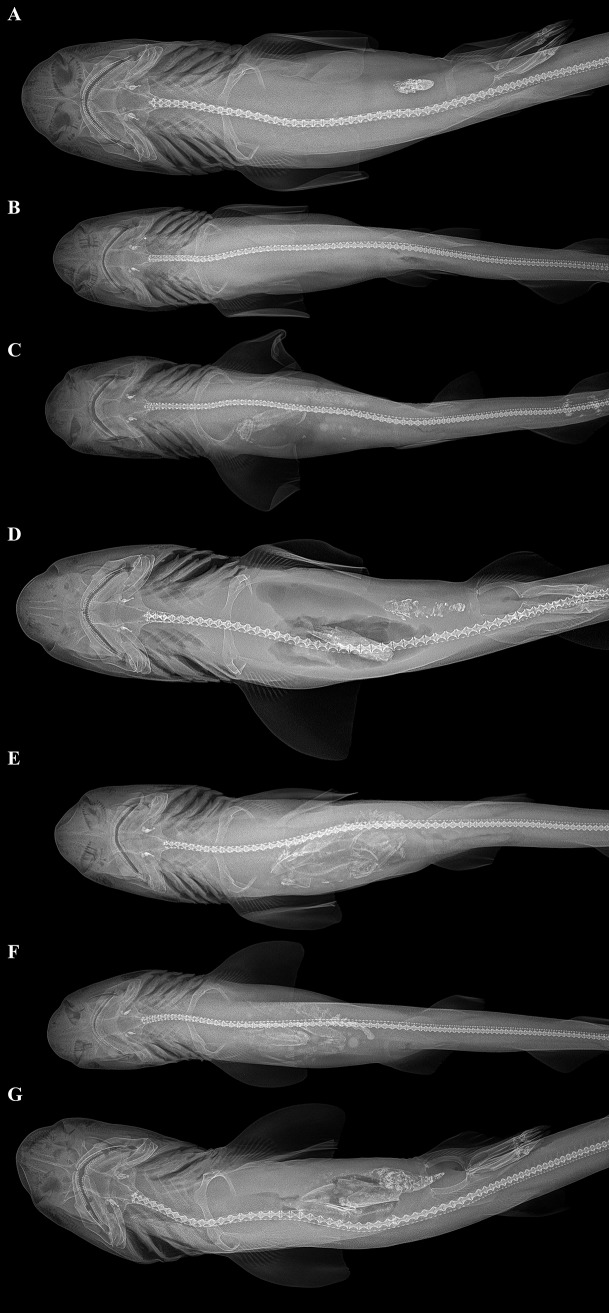
Exemplary radiographs of selected paratypes of *Bythaelurus stewarti* n. sp. in dorsal views depicting different scores of stomach fullness. (A) paratype, ZMH 26254, adult male, 391 mm TL, score 0, (B) paratype, ZMH 26254, juvenile female, 296.4 mm TL, score 0, (C) paratype, ZMH 26252, juvenile female, 299 mm TL, score 1 containing a teleost and few eye lenses, (D) paratype, ZMH 26254, adult male, 417 mm TL, score 1 containing a decapod (probably brachyuran) claw, (E) paratype, ZMH 26252, juvenile male, 340 mm TL, score 2 containing, among other remains, two decapod (probably brachyuran) claws and one teleost, (F) paratype, ZMH 26252, juvenile female, 299 mm TL, score 2 containing two decapod (probably brachyuran) claws and few eye lenses, and (G) paratype, ZMH 26254, adult male, 375 mm TL, score 2 containing a decapod (probably brachyuran) claw and a teleost.

The analysis of radiographs of all 121 type specimens of the new species revealed that 34 of 121 stomachs (28.1%) were empty (score 0), 78 (64.5%) were partially filled (score 1), and 9 (7.4%) were full or nearly full (score 2). Examples of the different scores can be found in Fig 15. The present findings are in line with those of other studies on different *Bythaelurus* spp. Nevertheless, the actual number of empty stomachs might be lower in the present study as soft food remains generally do not depict well in radiographs. In a study on *B*. *canescens* (Günther; for holotype data see [17]) by Acuña & Villarroel [18], the number of stomachs with food was 312 of a total of 513 stomachs examined (61%), accordingly the number of empty stomachs was 201 (39%). In a study by Lopez *et al*. [19], 37 of 50 stomachs of *B*. *canescens* sampled (74%) contained food, correspondingly 13 stomachs (26%) were empty. For *B*. *hispidus* Nair & Appukuttan [20] reported that 57 of 241 examined stomachs (24%) were empty. Nevertheless, in the same paper the authors indicated 52 of 241 empty stomachs (22%) when differentiating between adults and juveniles. Here, 36 stomachs showed traces of food, 60 stomachs were quarter full, 43 stomachs were half full, 17 three fourths full, 23 were full, and 10 gorged with food. Of 121 stomachs of *B*. *hispidus* examined by Akhilesh *et al*. [21], 24% were empty, 18% with trace contents only, 15% half full, 21% three quarters full, and 22% full.

**Fig 16:**
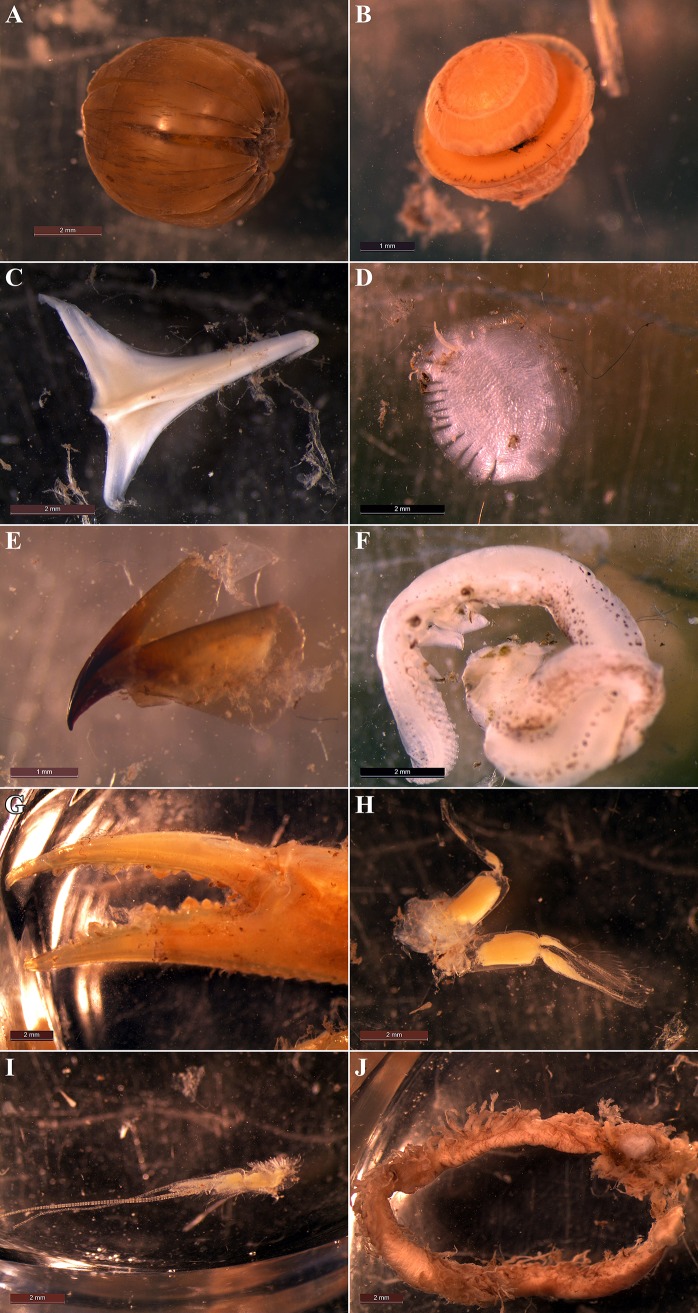
*Bythaelurus stewarti* n. sp., paratype, ZMH 26252, female, 295 mm TL, stomach contents. (A) partially digested teleost fish eye, (B) cephalopod eye, (C) probably a teleost bone but possibly a lophophore structure instead, (D) teleost scale, (E) beak of cephalopod *Ancistrocheirus* cf. *lesueurii*, (F) arm of cephalopod *Ancistrocheirus* cf. *lesueurii*, (G) decapod (probably brachyuran) claw, (H) antennae of a swimming decapod of the paraphyletic suborder “Natantia”, (I) antennulae of a swimming decapod of “Natantia”, (J) unidentified food remains, possibly a partially digested shrimp stomach.

In the present study, the diet mostly consisted of teleosts, cephalopods, and decapods. This is evidenced by the findings from radiographs, as well as the results of the dissection of female paratype 295 mm TL, ZMH 26252. The components most commonly found in radiographs, as well the dissected stomachs were partially digested teleost fish eyes (Fig 16, A). However, in radiographs teleost eyes and cephalopod eyes (Fig 16B) might be mixed up due to similar appearance. Based on radiographs, the frequency of occurrence of roundish structures, likely teleost eyes but possibly–to a lesser extent–cephalopod eyes, was 53.6%. Further teleost remains detected were parts of vertebral columns (Fig 15C, 15E and 15G), which were detected in radiographs only, other skeletal parts (Fig 16C), which were detected in both radiographs and the dissected stomach, as well as scales (Fig 16D), which were detected in the dissected stomach only. Based on radiographs, the frequency of occurrence of skeletal parts of teleosts was 20.0%. In addition to eyes, beaks (Fig 16E) and arms (Fig 16F) of cephalopods were also found in the dissected stomach, soft parts like arms probably being hardly detectable in radiographs. The beaks and arms were identified as *Ancistrocheirus* cf. *lesueurii* (D’Orbigny). With respect to decapods, different body parts were found in the dissected stomach (Fig 16G–16I), but in radiographs mainly claws or parts of claws were detected. The other crustacean parts are probably hard to detect in radiographs. The antennae and antennulae were likely from swimming decapods of the paraphyletic suborder “Natantia”, the claws were probably from brachyurans. In the radiographs, the frequency of occurrence of decapod remains was 14.5%. Another diet possibly ingested by the new species but hardly detectable in radiographs and not found in the examined specimens are polychaetes. Nevertheless, polychaetes were not found to be important food items in other *Bythaelurus* species, with only one ([18]) of two ([18,19]) studies on *B*. *canescens* reporting very low numbers of polychaetes and none of three ([20–22]) studies on *B*. *hispidus* detecting any polychaetes. Therefore, it is conceivable that polychaetes are not or hardly ingested by the new species. In the current study, based on radiographs the frequency of occurrence of unidentified stomach contents was 11.8%, one large example of unidentified food remains found in the dissected stomach is shown in Fig 16J, possibly representing a partially digested shrimp stomach.

The composition of prey items found in the current study is in line with results of earlier studies on species of *Bythealurus*: based on the percent index of relative importance (%IRI) Acuña & Villarroel [18] found crustaceans to be the most important prey item in *B*. *canescens* (~64%), followed by teleosts (~23%), unidentified digested remains (13.2%), cephalopods (<0.2%) and polychaetes (<0.1%). A different diet was reported by Lopez *et al*. [19] for *B*. *canescens*, who used a generalized index of food (%GI) and found siphonophores to be the most important prey item (68%), followed by cephalopods (20%) and teleosts (12%).

In the geographically and morphologically closest species, *B*. *hispidus*, Nair & Appukuttan [20] in a study on specimens caught from the Gulf of Mannar, India, detected that 61% of diet by volume (%V) was composed of teleosts, followed by cephalopods (18%), crustaceans (16%), mud (6%), gastropods (0.2%), and algae (0.1%). In their study, in adult specimens fish dominated in the food, followed by cephalopods and crustaceans, whereas in juveniles crustaceans ranked first, followed by fish, while cephalopods were not found. In a more recent study on the diet of *B*. *hispidus* by Akhilesh *et al*. [21], the analysis of stomach contents (%IRI) revealed teleosts as the primary diet (52%), followed by crustaceans (37%) and gastropods (1%).

### Parasites

**Fig 17:**
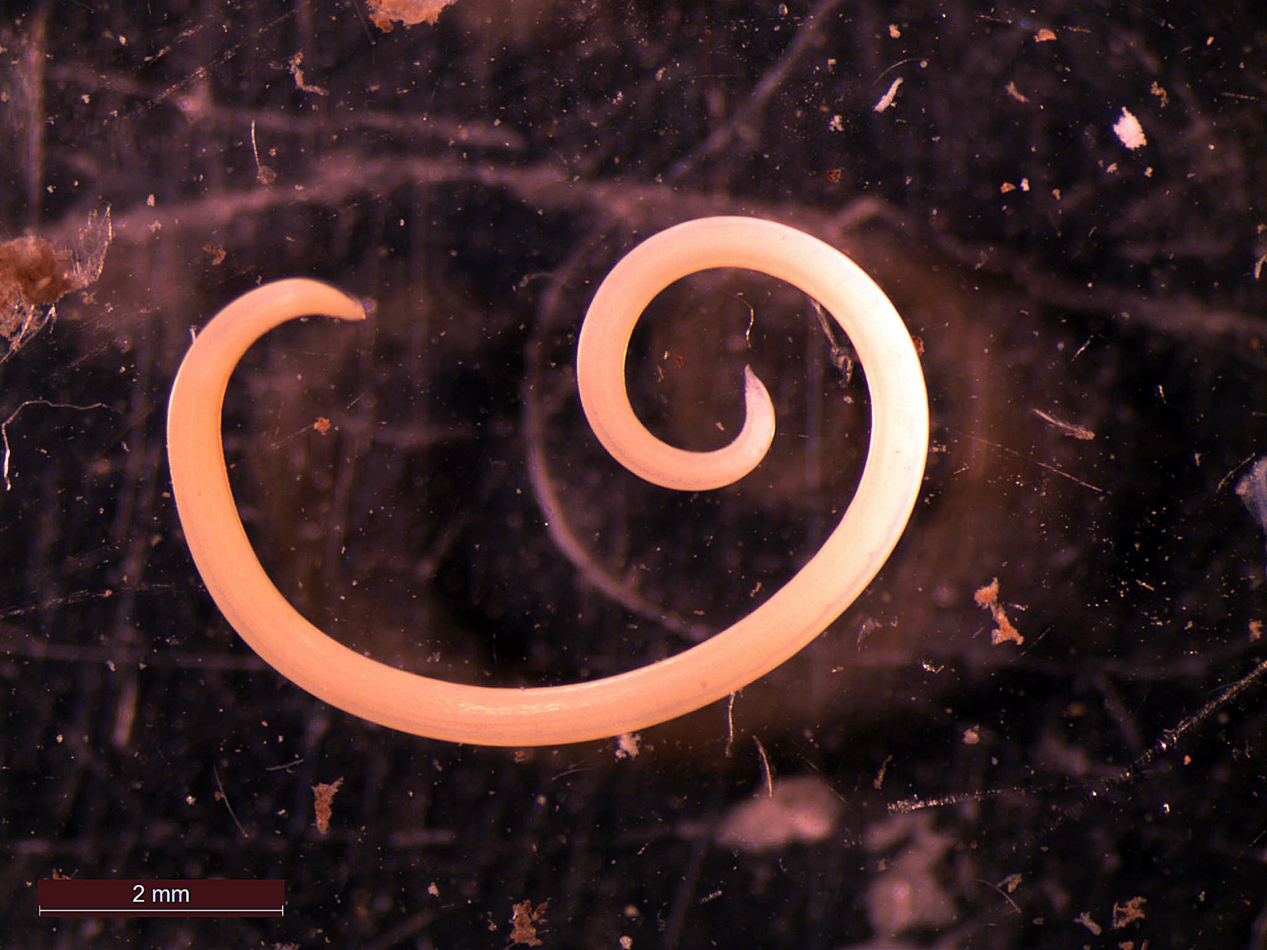
Ascaridid nematode dissected from the stomach wall of *Bythaelurus stewarti* n. sp., paratype, ZMH 26252, female, 295 mm TL.

Numerous nematodes were found in the stomach walls of two partially dissected paratypes (Fig 17). Based on their morphology they have been identified as members of the family Ascarididae. Ascaridid nematodes have previously been reported from other catshark genera, e.g. [23,24].

### Remarks

The reproductive mode was determined to be yolk-sac viviparous based on embryos found in adult female paratype ZMH 26253. Both uteri were filled with one near-term embryo each. The embryos both were females with 137.3 and 145.3 mm TL, respectively. The embryos were each enclosed in a thin, membranous egg case *in utero*. Yolk-sac viviparity is the rarest and most advanced reproductive mode within the Scyliorhinidae *sensu lato*, in which three reproductive modes occur [21]. In this reproductive mode females give birth to live young from a thin egg case *in utero* without direct link between embryo and mother via a uterine connection [21].

With regard to the apparent difference in maturity and maximum sizes between *B*. *stewarti* n. sp. and *B*. *hispidus*, intraspecific variation and phenotypic plasticity, caused by different environmental characteristics should be taken into consideration. According to Pitcher & Hart [25], individuals have evolved the capacity to overcompensate reproductive output in the face of variability in annual mortality. Compensation may result from changes in individual growth rates, age and size of maturation, reproductive output, and survival. One example is the spiny dogfish, *Squalus acanthias* Linnaeus, that has experienced reductions in neonate size, fecundity, age and size at maturity after decades of exploitation in the northwestern Atlantic [26,27]. However, the various further differences between *B*. *stewarti* n. sp. and *B*. *hispidus* warrant the description as a new species.

As catsharks of the genus *Bythaelurus* are generally inactive, demersal sharks [28,29], it is assumed that the new species from the Error Seamount is geographically isolated. Although other seamounts exist in the wider radius of the Error Seamount, they are usually located in much deeper waters, where *B*. *stewarti* n. sp., *B*. *hispidus* and similar species apparently do not occur. Furthermore, a deep channel of almost 4000 m depth separates the Error Seamount from the Socotra Islands. Therefore, the new species is supposed to be endemic to the Error Seamount, arisen from a speciation process, potentially driven by special environmental conditions and prey availability on the seamount. The assumption of the presence of an isolated population of *Bythaelurus* catsharks on the Error Seamount is supported by the fact that high levels of endemism are generally assumed for seamounts [30–35]. However, sampling biases might be caused by the occasional sampling of rare but widespread species [36,37]. Furthermore, the complex effects that seamounts have on ocean circulation and, accordingly, deposition of sediment and organic matter are poorly understood due to the large diversity in seamount size, shape, and distribution [30,35]. Nevertheless, as a result of their sharp relief across the floor of the ocean basins, seamounts generally have strong influences on water movements [33]. Processes like trapping of plankton (trophic focusing) and increased primary productivity resulting from localized upwelling generally cause seamounts to harbor richer benthic communities than surrounding deep-sea habitats [30,38]. The available rich food resources might possibly favor the growth of the specimens on the Error Seamount to larger sizes than all known populations of *B*. *hispidus*. As the Error Seamount is a flat-topped guyot, it probably has a rather quiescent summit, covered with soft sediment, and steep slopes of mostly bare rock following Clark *et al*. [35], possibly supporting the development of rich benthic communities. Nevertheless, values of bottom temperature and salinity, taken during cruise 17 of RV “Vityaz” and kindly provided by Sergei A. Evseenko and Aleksej V. Mishin (IORAS, P.P.Shirshov Institute of Oceanology of the Russian Academy of Sciences, Moscow), as well as M.F.W. Stehmann are very similar at the catch locations of the new species as compared to the catch locations of *B*. *hispidus*.

Comparative molecular analyses of *B*. *stewarti* n. sp. and *B*. *hispidus* are desirable for further clarification of the phylogenetic relationships and degree of differentiation. For this purpose, Thomas Knebelsberger (DZMB) kindly tried to perform molecular analyses from tissue samples taken internally from 10 paratypes of the new species and 14 specimens (13 specimens collected during cruise 17 of RV “Vityaz” plus specimen BMNH 1898.7.13.21) of *B*. *hispidus*. Unfortunately, these attempts were not successful due to formaldehyde-fixation of the “Vityaz” material and possibly due to long-time storage of the BMNH specimen. Although non-formaldehyde-fixed material of *B*. *hispidus* from other regions has become available (see [4]), non-formaldehyde-fixed specimens of the new species are not available, unfortunately, and may well not become available for a long time due to the remote type location.

## Discussion

*Bythaelurus stewarti* n. sp. differs from all congeners in the distribution, which is apparently restricted to the Error Seamount. It further differs from all congeners in a higher spiral valve turn count (11–12 vs. 6–10) and in the morphology of the branchial, trunk and lateral caudal-fin dermal denticles, which are loosely-spaced and not overlapping even in adult specimens of the new species, whereas they are closely-set and overlapping in adults of all other *Bythaelurus* species.

The new species is morphologically and geographically closest to *B*. *hispidus*. The most obvious difference of the new species compared to *B*. *hispidus* is the size: in *B*. *stewarti* n. sp., adult males range between 389 and 435 mm TL and juvenile males between 162 and 348 mm TL (subadult males and male embryos unknown). In contrast, in *B*. *hispidus* specimens from the geographically closest and in the comparative material by far best represented location Socotra Islands (n = 100), adult males have total lengths between 274 and 338 mm, subadult males between 254 and 285 mm TL, and juvenile males between 166 and 266 mm TL (one male embryo has 126.7 mm TL 70% ethanol preserved). From off the Andaman Islands, even smaller adult males (BMNH 1898.7.13.21 and USNM 221384) of *B*. *hispidus* with only 258.5 and 260 mm TL, respectively, and from the Gulf of Mannar, an even smaller subadult male specimen (SMF 11465) with only 212.9 mm TL were examined. These values are in line with specimens from southern India studied by Akhilesh *et al*. [21], who found that most males between 200 and 230 mm TL possessed partially calcified claspers, the smallest mature male had 228 mm TL and the largest immature male 265 mm TL. In this study, the maturity size (95% C.I.) of males was 235 (223–245) mm TL. In addition, the fresh ground color is darker in the new species and the ventral head usually bears dark grayish-brown mottling (vs. mostly uniformly yellowish or whitish in *B*. *hispidus*). Distinct differences can be found in the morphology of the dermal denticles: in adult specimens of the new species, the snout denticles are broader and have less pronounced ridges, the denticles in branchial area, on lateral trunk and on lateral caudal fin are smaller, less elongate and set much less densely and their surface is very strongly and fully structured by reticulations (vs. structured by reticulations only in basal fourth) and the denticles on anterior dorsal caudal-fin are less elongate and set slightly more loosely. When comparing the denticles of juveniles of the new species with those of *B*. *hispidus* specimens with similar total lengths, even more pronounced differences become apparent: the snout denticles of juvenile *B*. *stewarti* n. sp. are strongly structured by reticulations, stouter in shape and have less pronounced ridges, the denticles in branchial area, on lateral trunk and on lateral caudal fin are even less densely set and have even smaller lateral cusps than in adults, and the denticles on the anterior dorsal caudal-fin margin are similar to the trunk denticles of adults, leading to strong differences compared to *B*. *hispidus*. Another distinct difference between *B*. *stewarti* n. sp. and *B*. *hispidus* can be found in the clasper morphology: the claspers of the new species are more slender and gradually narrowing to the bluntly pointed tip without knob-like apex (vs. claspers broader and with distinct knob-like apex). Furthermore, the new species has more spiral valve turns (11–12 vs. 8–10) and numerous differences in morphometrics, particularly in preoral length, caudal-fin postventral margin, pelvic height, tail height at pelvic base end, pelvic anterior margin of both sexes, and total length and clasper base width of adult males, are evident statistically.

*Bythaelurus stewarti* n. sp. can easily be distinguished from *B*. *bachi* by the body shape (slender vs. stout), coloration (blotched dorsally and mottled ventrally vs. plain beige to light grayish-brown), tooth morphology (teeth in upper and lower jaws tricuspidate with median cusp much longer than lateral cusps vs. posterolateral upper and antero- and posterolateral lower jaw teeth tetra- to pentacuspidate and with median cusp only slightly longer than lateral cusps), absence (vs. presence) of composite oral papillae, low (vs. high) diversity in dermal denticle morphology, absence (vs. presence) of embryonic enlarged, blunt, spatulate, cross-based denticles on dorsal anterior trunk, trunk shorter than tail (vs. trunk about as long as tail), apex of pelvic fins narrowly (vs. very broadly) rounded, first dorsal fin higer than second one (vs. subequal in height), apex and free rear tip of first dorsal fin angular (vs. rounded), pelvic—anal space longer (8.0–12.7% TL vs. 3.0–8.6% TL and 0.5–0.8 vs. 0.1–0.4 times pectoral—pelvic space), apex of anal fin angular (vs. rounded), anal-fin base length 2.6–6.0 (vs. 1.4–2.9) times fin height and 1.4–2.3 (vs. 1.0–1.5) times second dorsal-fin base length, claspers terminating distinctly before anal-fin origin (vs. overlapping) and very slender vs. broad (base width 1.4–1.5% TL vs. 2.3–3.1% TL), with (vs. without) clasper hooks and component envelope overlapping (vs. not overlapping) part of clasper groove, more spiral valve turns (11–12 vs. 6–8), reproductive mode (yolk-sac viviparous vs. oviparous), and shallower occurrence (380–420 m vs. 910–1365 m depth).

Compared to *B*. *clevai*, the new species differs in the dorsal coloration consisting of few large dark blotches of similar size (vs. numerous small and large dark blotches), a shorter snout (preorbital length 4.9–7.4% TL vs. 8.3% TL, preoral length 4.5–6.7% TL vs. 7.0% TL), shorter pre-outer (1.7–3.7% TL vs. 4.3% TL) and pre-inner (3.5–5.7% TL vs. 6.1% TL) nostril lengths, shorter upper labial furrows (0.7–1.6% TL vs. 1.8% TL), longer eyes (2.9–4.5% TL vs. 2.8% TL), a smaller ratio eye length in predorsal space (10.2–15.5 vs. 16.7), a longer intergill length (3.8–7.1% TL vs. 3.3% TL), a shorter anal—caudal space (0–1.9% TL vs. 2.8% TL), more slender and longer claspers (base width 1.4–1.5% TL vs. 1.8% TL, inner margin length 10.1–11.3% TL vs. 8.9% TL) without (vs. with) knob-like apex, and fewer diplospondylous (37–45 vs. 46) and total (75–84 vs. 88) precaudal vertebrae.

The new species differs from *B*. *lutarius* (Springer & D’Aubrey), e.g., in the presence of numerous oral papillae on tongue and roof of mouth (vs. papillae virtually absent or absent), coloration (blotched dorsally and mottled ventrally vs. plain-colored except for dusky markings near the bases of the dorsal fins), a broader anterior head (width at posterior edge of nostrils 9.3–11.0% TL vs. 8.8% TL, width at mouth corners 10.3–13.3% TL vs. 9.4% TL), shorter pre-first (41.8–47.6% TL vs. 47.7% TL) and pre-second (57.5–65.1% TL vs. 65.9% TL) dorsal-fin lengths, a shorter pre-anal fin length (51.4–60.2% TL vs. 60.4% TL), a shorter pelvic—anal space (8.0–12.7% TL vs. 13.6% TL and 0.3–0.8 vs. 0.9 times pectoral—pelvic space), a shorter anal—caudal space (0–1.9% TL vs. 2.4% TL), a more slender caudal peduncle (width 1.8–2.9% TL vs. 1.5% TL), a longer (length 28.2–34.4% TL vs. 26.6% TL) and higher (height 5.3–7.3% TL vs. 5.0% TL, terminal lobe height 2.1–3.7% TL vs. 4.0% TL) caudal fin, longer claspers (inner margin length 10.1–11.3% TL vs. 9.6% TL), and more spiral valve turns (11–12 vs. 10).

*Bythaelurus stewarti* n. sp. can easily be distinguished from *B*. *naylori* by the body shape (slender vs. stout), coloration (dorsally dark grayish-brown with dark blotches at nape, on flank between pectoral and pelvic fin, dorsolaterally on body below first and second dorsal fins, and one across caudal fin, ventrally grayish-white with dark grayish-brown mottling on head vs. medium to dark brown with light-edged fins and distinctly dark, dusky-colored snout), presence (vs. absence) of oral papillae on tongue and roof of mouth, only slightly (vs. distinctly) enlarged dermal denticles on the anterior dorsal caudal-fin margin, trunk shorter than tail (vs. trunk about as long as or slightly longer than tail), first dorsal fin higer than second one (vs. subequal in height), apex of anal fin angular (vs. rounded), anal-fin base length 2.6–6.0 (vs. 1.5–2.7) times fin height and 1.4–2.3 (vs. 1.1–1.5) times second dorsal-fin base length, claspers terminating distinctly before anal-fin origin (vs. overlapping) and very slender vs. broad (base width 1.4–1.5% TL vs. 2.0–2.2% TL), more spiral valve turns (11–12 vs. 7–8), reproductive mode (yolk-sac viviparous vs. oviparous), smaller maturity and maximum sizes (maturity size 35–39 cm TL, maximum size 44 cm TL vs. maturity size 38–48 cm TL, maximum size 55 cm TL), and shallower occurrence (380–420 m vs. 752–1443 m depth).

In contrast to *B*. *tenuicephalus*, the new species has a broad head with distinct lateral indention (vs. head slender without distinct lateral indention), a broader anterior head (width at level of lateral indention 6.4–8.0% TL vs. 6.0–6.1% TL, width at maximum outer extent of nostrils 8.1–10.1% TL vs. 7.2–7.3% TL, width at posterior edge of nostrils 9.3–11.0% TL vs. 8.4–8.5% TL), more slender and longer claspers (base width 1.4–1.5% TL vs. 2.1% TL, outer margin length 6.3–8.5% TL vs. 5.8% TL, inner margin length 10.1–11.3% TL vs. 9.1% TL) of narrowly rod-like shape (vs. broad stem and proximal glans which distally rapidly tapers to the narrow tip), larger maturity and maximum sizes (maturity size 35–39 cm TL, maximum size 44 cm TL vs. maturity size ~28 cm TL, maximum size ~30 cm TL), more spiral valve turns (11–12 vs. 7 in the holotype of *B*. *tenuicephalus*), and shallower occurrence (380–420 m vs. 463–550 m depth).

*B*. *stewarti* n. sp. differs from *B*. *vivaldii* in the coloration (dorsally few large dark blotches of similar size vs. reportedly 8–9 dark, broad but inconspicuous transverse bars), presence of numerous, densely-set (vs. few) papillae on the tongue, tooth morphology in upper jaw (anterolateral teeth with median cusp much longer than lateral cusps vs. lateral cusps relatively larger and median cusp relatively smaller, leading to a reduced size difference between median and lateral cusps), tooth morphology in lower jaw (anterolateral teeth tricuspidate with median cusp much longer than lateral cusps vs. tri- to pentacuspidate with median cusp only slightly longer than lateral cusps), morphology of snout dermal denticles (denticles with two to four vs. four to six narrow ridges), morphology of dermal denticles in branchial area, on lateral trunk and on lateral caudal fin (denticles loosely set, not overlapping and surface very strongly and fully structured by reticulations vs. denticles densely set und structured by reticulations in basal third only), morphology of dermal denticles on the anterior dorsal caudal-fin margin (denticles densely set and with very strong and pronounced lateral ridges in adults, denticles loosely set and surface very strongly and fully structured by reticulations in juveniles vs. denticles densely set and with indistinct lateral ridges), apices of first dorsal and anal fins angular (vs. rounded), shorter dorsal precaudal (67.6–73.9% TL vs. 75.1–78% TL), ventral precaudal (66.1–71.8% TL vs. 73.5–73.9% TL), pre-second dorsal fin (57.5–65.1% TL vs. 67.0–67.9% TL) and pre-first dorsal fin (41.8–47.6% TL vs. 48.2–49.6% TL) lengths, a shorter pre-anal fin length (51.4–60.2% TL vs. 60.2–62.5% TL), a longer second dorsal-fin anterior (7.0–11.5% TL vs. 5.1–7.0% TL) but shorter posterior (1.4–3.3% TL vs. 3.5% TL) margin, shorter pelvic-fin total length (7.0–10.8% TL vs. 10.7–11.5% TL) and posterior margin (3.2–6.7% TL vs. 7.5–7.6% TL), a distinctly longer caudal fin (length 28.2–34.4% TL vs. 23.8–24.4% TL) with shallower terminal lobe (height 2.1–3.7% TL vs. 5.0% TL), a narrower mouth (width 4.6–8.8% TL vs. 9.2–9.6% TL), shorter lower labial furrows (length 1.0–2.3% TL vs. 2.3–2.4% TL), more spiral valve turns (11–12 vs. 7), larger maturity and maximum sizes (maturity size 35–39 cm TL, maximum size 44 cm TL vs. maturity size ~30 cm TL, maximum size ~35 cm TL), presumably reproductive mode (yolk-sac viviparous vs. presumably oviparous), and shallower occurrence (380–420 m vs. 628 m depth).

A detailed comparison of the new species with *B*. *alcockii* (Garman) is not possible due to the lack of specimens or detailed descriptions. That species was described from the Arabian Sea without exact locality data and is known only from the holotype, which has been lost [28] (K.V. Akhilesh, pers. comm., 2014). Following Alcock’s [39] description of the lost holotype of *B*. *alcockii*, *B*. *stewarti* n. sp. has a strongly different coloration (blotched dorsally and mottled ventrally vs. blackish with hoary gray surface and some fins white-tipped posteriorly in *B*. *alcockii*) and tooth morphology (teeth in upper jaw tricuspidate with median cusp much longer than small lateral cusps, teeth in lower jaw similar to those of upper jaw but with larger lateral cusps vs. teeth with median and lateral cusps of subequal length). Furthermore, the new species was caught in much shallower water (380–420 m vs. 1134–1262 m depth). *B*. *alcockii* was originally described as *Halaelurus alcockii* and preliminarily placed in the subgenus *Bythaelurus* by Compagno [3]. However, the dark coloration and long snout combined with great catch depth, reported for *B*. *alcockii* by Alcock [39], indicate that this species possibly belongs to *Apristurus* rather than to *Bythaelurus*, like assumed by Compagno [28]. However, the tooth morphology described for *B*. *alcockii* by Alcock [39] does not fit the characters of neither *Bythaelurus* nor *Apristurus* species and therefore seems questionable. Overall, the validity of the species is questionable [1,3–5,9,28,29]. As the only known specimen has been lost, it is currently impossible to resolve this issue and *B*. *alcockii* remains a species of uncertain validity and generic assignment.

### Review of *Bythaelurus* species

**Fig 18:**
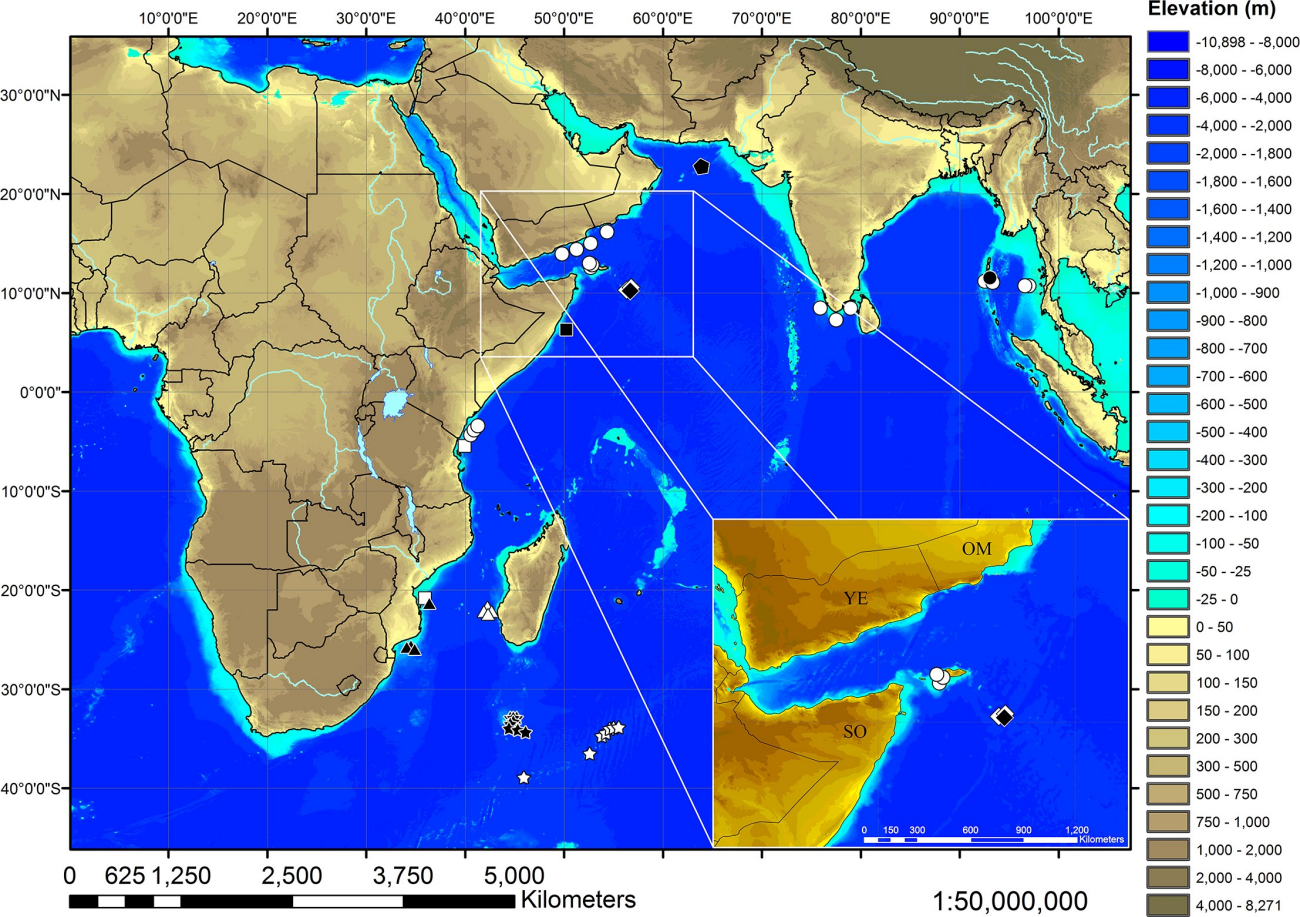
Map of the Indian Ocean depicting the verified occurrences of nine species of *Bythaelurus* in the Indian Ocean. The occurrences are based on examined material except for *B*. *clevai* (based on one examined specimen plus catch locations of the type specimens taken from Séret [12] and *B*. *alcockii* (no specimen available, catch location of the lost holotype indicated as Arabian Sea in Garman [40]). *Bythaelurus alcockii*: black pentagon, *B*. *bachi*: black stars, *B*. *clevai*: white triangles, *B*. *hispidus*: black (holotype) and white (other specimens) circles, *B*. *lutarius*: black triangles, *B*. *naylori*: white stars, *B*. *stewarti* n. sp.: black and white diamonds, *B*. *tenuicephalus*: white squares, *B*. *vivaldii*: black square. Inset of the Gulf of Aden area depicts the catch locations of the holotype (black diamond) and paratypes (black and white diamonds) of *Bythaelurus stewarti* n. sp. on Error Seamount and catch locations of 100 comparative specimens of *B*. *hispidus* from off the Socotra Islands (white circles). Country abbreviations follow ISO 3166–1 (OM = Oman, SO = Somalia, YE = Yemen).

The 14 (including *B*. *stewarti* n. sp.) currently valid species of *Bythaelurus* are small to medium-sized catsharks (maximum total lengths from 30 cm to 76 cm) found in deep water on continental and insular slopes in temperate and tropical latitudes of the Indian and Pacific oceans, between depths of 200 and 1443 m [1,4,5]. Geographically, the western Indian Ocean appears to be a hotspot of *Bythaelurus* species diversity with nine of the 14 currently valid species occurring in this area. A map showing the verified occurrences of nine of the 10 Indian Ocean species of *Bythaelurus* can be found in Fig 18 (the type location of *B*. *incanus* Last & Stevens is not depicted). Eight of the nine species in the map, i.e. *B*. *alcockii*, *B*. *bachi*, *B*. *clevai*, *B*. *lutarius*, *B*. *naylori*, *B*. *stewarti* n. sp., *B*. *tenuicephalus* and *B*. *vivaldii* are found exclusively in the western Indian Ocean, whereas the nineth species, *B*. *hispidus*, is also known from the eastern Indian Ocean [1]. Nevertheless, intrageneric differences in general morphology and body shape, the presence or absence of oral papillae, the presence or absence of a crest of enlarged dermal denticles on the anterior dorsal caudal-fin margin, and, particularly, genetics and reproductive modes indicate that *Bythaelurus* possibly comprises species from at least two different genera.

The 14 currently valid species can be grouped into two general morphotypes: one consists of species with slender bodies, i.e. *Bythaelurus clevai*, *B*. *hispidus*, *B*. *lutarius*, *B*. *stewarti* n. sp., and *B*. *tenuicephalus*, the other includes species with stocky bodies, at least in large specimens, i.e. *B*. *bachi*, *B*. *canescens*, *B*. *dawsoni* (Springer), *B*. *giddingsi* McCosker, Long & Baldwin, *B*. *immaculatus* (Chu & Meng), *B*. *incanus*, *B*. *naylori*, and *B*. *vivaldii*. The body shape of *B*. *alcockii* is unknown.

Another possible grouping arises from the presence or absence of oral papillae: species with numerous oral papillae are *B*. *bachi*, *B*. *canescens*, *B*. *clevai*, *B*. *dawsoni*, *B*. *giddingsi*, *B*. *hispidus*, possibly *B*. *immaculatus*, *B*. *incanus*, *B*. *stewarti* n. sp., *B*. *tenuicephalus*, and *B*. *vivaldii*, species without (or with rudimentary) oral papillae are *B*. *lutarius* and *B*. *naylori*. The presence of oral papillae in *B*. *immaculatus* is unknown but it is assumed that the species has oral papillae based on its apparently close morphological relationship to *B*. *canescens* and *B*. *incanus*. White & Last [41] did not examine the holotype of *B*. *immaculatus* for the presence of oral papillae (W.T. White, pers. comm., 2015). Furthermore, none of the type specimens could be found upon recent requests (H.-C. Ho & X.-Y. Kong, pers. comm., 2015 & 2016) so the whereabouts of the type specimens and the presence of oral papillae remain unclear. The presence of oral papillae in *B*. *alcockii* is unknown.

A third possible grouping arises from the reproductive modes of *Bythaelurus* species that was reviewed by Francis [42]. He noted that some species, e.g. *B*. *canescens* and *B*. *dawsoni*, are oviparous, whereas others are viviparous.

A detailed comparison of morphological and morphometric characteristics, as well as maturity and maximum sizes of all valid species of *Bythaelurus* can be found in Table 5, an overview of geographic and depth distributions, reproductive modes and meristic characteristics of the 14 species of *Bythaelurus* is shown in Table 6.

**Table 5:** Selected morphological and morphometric characteristics of all valid *Bythaelurus* species.

Species	Common name	Coloration	Labial furrows	LLA/ULA	ANB/D2B	VCL/SVL	POB/EYL	IDS/D1B	PPS/IDS	Caudal crest	Maturity size	Maximum size	Remarks
*Bythaelurus alcockii* (Garman, 1913)	Arabian Catshark	blackish with hoary gray surface and some fins white-tipped posteriorly	unknown	unknown	unknown	unknown	unknown	unknown	unknown	unknown	unknown	unknown	validity and generic assignment questionable [1,3–5,9,28,29,43–47]
*Bythaelurus bachi* Weigmann, Ebert, Clerkin, Stehmann & Naylor, 2016	Bach's Catshark	plain beige to light grayish-brown	distinct	1.1–2.8^44^	1.0–1.5^44^	0.9–1.4^44^	0.9–2.0^44^	1.0–1.8^44^	1.5–3.2^44^	no	36–40 cm TL	45 cm TL	easily recognized by the bright coloration, stocky body, high diversity in dermal denticle morphology and presence of composite oral papillae
*Bythaelurus canescens* (Günther, 1878)	Dusky Catshark	plain dark brown or blackish above and below	distinct	1.7–3.0^4^	1.1–1.3^4^	1.3^1^	~0.9–1.3^41^	1.4–1.8^4^	1.5–2.2^41^	no	52–59 cm TL	73 cm TL	type species of *Bythaelurus*; common bycatch in demersal trawl and longline fisheries in central and southern Chile [48]; large and plain dark species with a stocky body in large specimens
*Bythaelurus clevai* (Séret, 1987)	Broadhead Catshark	grayish with a pattern of dark brown saddle-like markings on back and tail, with variegated dark brown blotches on flanks, grayish with brown speckles inside of the mouth, ventral surface whitish	distinct	1.0–~1.3^16^	~1.4–1.8^16^	~1.1–~1.5^16^	~2.4–2.9^16^	2.0–2.3^16^	1.1–1.7^16^	no	28–36 cm TL	40 cm TL	easily recognized by the distinctive color pattern
*Bythaelurus dawsoni* (Springer, 1971)	New Zealand Catshark / Dawson's Catshark	light brown to gray on dorsal and lateral surfaces with a line of qhite spots on sides of small individuals and whitish ventrally, fin tips with broad white areas and caudal fin with dark bands	distinct	1.6–4.5^10^	~1.2–1.5^10^	~1.1–1.5^10^	1.1–1.8^10^	1.4–1.6^10^	1.6–2.1^10^	no	32–38 cm TL	42 cm TL	easily recognized by the distinctive color pattern, particularly in juveniles and presence of only a few papillae on roof of mouth and tongue
*Bythaelurus giddingsi* McCosker, Long & Baldwin, 2012	Galápagos Catshark / Jaguar Catshark	chocolate brown dorsally with pale spots, the largest being about equal in size to eye diameter above midline, smaller below; posterior margin of dorsal, pectoral, and pelvic fins pale; ventral surface pale	distinct	1.9–2.9^7^	1.0–1.3^7^	0.9–1.3^7^	0.4–1.3^7^	1.4–2.2^7^	1.4–2.0^7^	yes	~40–45 cm TL	>45 cm TL	easily recognized by the distinctive color pattern. Ratio POB/EYL of 0.4, calculated from the data in McCosker *et al*. [49] for paratype SAM-35042, appears to be erroneous (0.9–1.3 in other specimens). The holotype of 402 mm TL is listed as both, adult and appearing to be approaching sexual maturity in the original description
*Bythaelurus hispidus* (Alcock, 1891)	Bristly Catshark	ground color variable,brownish to grayish, 5–6 indistinct, dark blotches on trunk and tail, few specimens reported to be pale brown with gray bands, few to have white markings and several to be almost white with a few patches of pigmentation (unpublished data of comparative specimens and [50])	distinct	1.0–~2.5^117^	1.4–2.2^117^	1.1–1.4^117^	1.2–2.2^106^	0.7–2.4^117^	1.1–4.1^117^	no	21–28 cm TL in males	39 cm TL	one of the smallest and most widespread species of *Bythaelurus*, adult males 27–34 cm TL, usually to ~30 cm TL; adult claspers moderately broad and abruptly narrowing before the tip with knob-like apex
*Bythaelurus immaculatus* (Chu & Meng, 1982)	Spotless Catshark	plain dark yellowish brown	reduced	~1.0^1^	1.0^3^	<1.0^1^	~1.0^1^	<2.0^3^ or >2.2^1^	2.4–2.5^3^	no	<71 cm TL	76 cm TL	known only from the three adult type specimens; the largest species of *Bythaelurus*; length of lateral trunk denticles less than twice their width; IDS/D1B given as <2.0 by Chu & Meng [51] but as >2.2 by White & Last [41]
*Bythaelurus incanus* Last & Stevens, 2008	Dusky Catshark	mostly plain grayish-brown with a few white blotches on belly	reduced	0.8^1^	1.2^1^	1.3^1^	1.3^1^	1.7^1^	1.7^1^	no	>45 cm TL	>45 cm TL	known only from the juvenile male holotype; differs from *B*. *immaculatus* in some morphometrics, more elongate lateral trunk denticles (length more than twice their width) and the presence (vs. absence) of few white blotches on ventral belly [43]
*Bythaelurus lutarius* (Springer & D’Aubrey, 1972)	Mud Catshark / Brown Catshark	largely plain with dusky areas near the fins	distinct	1.1–1.8^41^	~1.7–~2.3^41^	~1.1–~1.3^17^	~1.8–~2.1^35^	~2.1–2.5^41^	1.2–~2.2^41^	no	28–31 cm TL	39 cm TL	easily recognized by the absence (or virtual absence) of oral papillae combined with a slender body and largely plain coloration; the slender claspers gradually narrow to the bluntly pointed tip without knob-like apex. Records of the species from off Somalia [44,52,53] are based on *B*. *tenuicephalus* and *B*. *vivaldii* [5]. One much larger specimen of 491 mm (SAIAB 6152) reported by Compagno [3] is actually *Halaelurus lineatus* [1]
*Bythaelurus naylori* Ebert & Clerkin, 2015	Dusky Snout Catshark	medium to dark brown with light fin edges and a distinct dark dusky-colored snout	distinct	1.4–2.4^10^	1.1–1.5^10^	0.8–1.0^10^	1.1–1.6^10^	1.2–1.7^10^	2.4–3.2^10^	yes	38–48 cm TL	55 cm TL	easily recognized by the dark dusky-colored snout, prominent crest of enlarged dermal denticles on the anterior dorsal caudal-fin margin, absence of oral papillae and stocky body
***Bythaelurus stewarti* n. sp.**	Error Seamount Catshark	dorsally dark grayish-brown with rather indistinct dark blotches at nape, on flank, below both dorsal fins, and across caudal fin; ventral side grayish-white, usually with dark grayish-brown mottling on head	distinct	0.8–2.2^120^	1.4–2.3^120^	1.2–1.5^120^	1.3–2.4^120^	1.3–2.3^120^	1.2–2.1^120^	no	35–39 cm TL	44 cm TL	easily distinguished from *B*. *hispidus* by the large size, high spiral valve counts, loosely-set branchial, trunk and lateral caudal-fin dermal denticles even in adults with a surface that is very strongly and fully structured by reticulations, and slender claspers that gradually narrow to the bluntly pointed tip without knob-like apex
*Bythaelurus tenuicephalus* Kaschner, Weigmann & Thiel, 2015	Narrowhead Catshark	bicolored with an abrupt lateral demarcation of medium brown dorsal and whitish ventral color, about five indistinct darker blotches on dorsolateral surface at gill slits and dorsal and caudal fins; fins lighter towards their margins	distinct	1.3–1.5^2^	1.7^2^	1.1–1.3^2^	2.0–2.3^2^	1.9–2.0^2^	1.5–1.6^2^	no	~28 cm TL	~30 cm TL	known only from the two type specimens; easily recognized by the narrow head without distinct lateral indention anterior to outer nostrils; adult claspers rather broad (base width ~2% TL) and without knob-like apex
*Bythaelurus vivaldii* Weigmann & Kaschner, 2017	Vivaldi's Catshark	reported to have a color pattern consisting of 8–9 dark, broad but inconspicuous transverse bars similar to *Halaelurus quagga* [5]	distinct	1.6–2.0^2^	1.7–1.9^2^	1.2–1.3^2^	1.9^2^	2.2–2.3^2^	1.1–1.3^2^	no	~30 cm TL	~35 cm TL	known only from the two type specimens; differs from all WIO congeners in the short caudal fin (length ~24% TL). Furthermore, it has a broad mouth (width ~9–10% TL), very few oral papillae on the tongue and a stocky body in large specimens

**Footer:** Superscript numbers in morphometric ratios indicate numbers of specimens on which the values are based. Data sources: data of *Bythaelurus stewarti* n. sp. from the present study, *B*. *bachi* from individual measurements of all type specimens, *B*. *canescens* from unpublished measurements of comparative material, [44], and [54], *B*. *clevai* from [9] and [12], *B*. *dawsoni* from unpublished measurements of comparative material, [44], and [55], *B*. *giddingsi* from [49], *B*. *hispidus* from unpublished measurements of comparative material, [9], [44], and [52], *B*. *immaculatus* from [41], [43], and [51], *B*. *incanus* from [43], *B*. *lutarius* from unpublished measurements of comparative material, [9], [44], [52], and [53], *B*. *naylori* from individual measurements of type specimens, *B*. *tenuicephalus* from [9], *B*. *vivaldii* from [5]. "~" indicates approximate values either based on calculations from minimum / maximum values (*B*. *canescens*, *B*. *clevai*, *B*. *dawsoni*, *B*. *hispidus*, *B*. *lutarius*), imprecise data (*B*. *immaculatus*) or estimations (*B*. *tenuicephalus* and *B*. *vivaldii*). Abbreviations: ANB = anal fin base length, D1B = D1 base length, D2B = D2 base length, EYL = eye length, IDS = interdorsal space, LLA = lower labial furrow length, POB = preorbital length, PPS = pectoral—pelvic space, SVL = snout—anterior vent length, ULA = upper labial furrow length, VCL = anterior vent—caudal tip length, Caudal crest = prominent crest of comb-like dermal denticles on dorsal caudal-fin margin.

**Table 6:** Geographic and depth distributions, reproductive modes, as well as tooth row, vertebral and spiral valve counts of all valid *Bythaelurus* species.

Species	Geographic distribution		Depth distribution	Reproductive mode	Tooth row counts		Vertebral counts					Spiral valve counts
	large-scale distribution	detailed distribution			upper jaw	lower jaw	monospondylous	diplospondylous precaudal	total precaudal	caudal	total	
*Bythaelurus alcockii*	WIO	Arabian Sea	1134–1262 m	unknown	unknown	unknown	unknown	unknown	unknown	unknown	unknown	unknown
*Bythaelurus bachi*	WIO	southern Madagascar Ridge	910–1365 m	single oviparity	70–84^14^	60–76^14^	38–43^28^	33–43^28^	73–83^28^	48–56^28^	124–132^28^	6–8^3^
*Bythaelurus canescens*	SEP	Peru and Chile to the Straits of Magellan	237–1260 m	single oviparity	80–101^2^	~60–111^2^	39–43^>20^	43^1^	82^1^	40^1^	117–132^24^	7–8^5^
*Bythaelurus clevai*	WIO	Madagascar	400–500 m	yolk-sac viviparity	62–80^16^	53–70^16^	39–45^16^	46–54^15^	88^1^	50^1^	127–142^14^	unknown
*Bythaelurus dawsoni*	SWP	New Zealand	240–992 m	single oviparity	~64–~66^2^	~62–~70^2^	36–38^11^	31–36^3^	67–73^11^	~55–~62^10^	~124–~129^10^	unknown
*Bythaelurus giddingsi*	SEP	Galapagos Islands	428–562 m	unknown	20–23^7^	23–26^7^	39–42^7^	40–44^7^*	81–85^7^	~40–~51^7^	~121–~134^7^	unknown
*Bythaelurus hispidus*	WIO, EIO	Kenya, Socotra Islands, Yemen, Oman, southern India, Andaman Islands and Myanmar	200–800 m	yolk-sac viviparity	67–70^2^	61–83^2^	35–38^14^	37–41^2^	74–78^2^	48^2^	122–131^14^	8–10^3^
*Bythaelurus immaculatus*	NWP	Hainan Island, South China Sea	534–1020 m	unknown	unknown	unknown	unknown	unknown	unknown	unknown	unknown	unknown
*Bythaelurus incanus*	EIO	Ashmore Terrace, western Australia	900–1000 m	unknown	~99^1^	~101^1^	46^1^	~47^1^	~93^1^	~49^1^	~142^1^	unknown
*Bythaelurus lutarius*	WIO	Mozambique	338–766 m	yolk-sac viviparity	74–76^2^	78–86^2^	38–42^29^	44–50^12^	82–89^18^	49^1^	129–142^46^	10^2^
*Bythaelurus naylori*	WIO	Southwest Indian Ridge	752–1443 m	single oviparity	>70^10^	>70^10^	36–41^10^	25–38^10^	64–78^10^	~44–~54^10^	~114–~128^10^	7–8^6^
***Bythaelurus stewarti* n. sp.**	WIO	Error Seamount	380–400 m	yolk-sac viviparity	64–85^50^	64–88^50^	37–42^121^	37–45^121^	75–84^121^	44–59^121^	125–140^121^	11–12^2^
*Bythaelurus tenuicephalus*	WIO	Tanzania and Mozambique	463–550 m	supposedly yolk-sac viviparity	67–76^2^	62–64^2^	38–40^2^	43–50^2^	83–88^2^	50^2^	133–138^2^	7^1^
*Bythaelurus vivaldii*	WIO	Somalia	628 m	supposedly single oviparity	68–75^2^	64^1^	38^2^	43–44^2^	81–82^2^	~54–~58^2^	~135–~140^2^	7^1^

**Footer:** Superscript numbers in meristics indicate numbers of specimens on which the values are based.

* In Table 2 of McCosker *et al*. [49], individual values for diplospondylous precaudal count indicate a maximum of 44, but range indicates 43 as maximum count. Data sources: data of *Bythaelurus stewarti* n. sp. from the present study, data of *B*. *bachi* from [4] and of *B*. *vivaldii* from [5]; sources for all other species: maximum total length and geographic distribution from [1] except for maximum total length of *B*. *hispidus* from the present study; depth data from [1] except for maximum depth of *B*. *canescens* from [54]; reproductive modes from [21], [42], [48], and [56]; counts from [3], [4], [5], [9], [12], [43], [44], [49], [52], [53], [55], and [56], as well as unpublished counts of comparative specimens of *B*. *canescens*, *B*. *dawsoni*, and *B*. *tenuicephalus*. Large-scale distribution indicates distribution across 10 major ocean areas as defined by Weigmann [1]: EIO = eastern Indian Ocean, NWP = northwestern Pacific Ocean, SEP = southeastern Pacific Ocean, SWP = southwestern Pacific Ocean, WIO = western Indian Ocean. Sources for detailed distribution of *B*. *hispidus*: Kenya, Socotra Islands: comparative material examined; Yemen: [57]; Oman: L. Jawad, pers. comm., 2013; southern India: comparative material examined, [20–22,50,58]; Andaman Islands: comparative material examined, [9,12,44,52,59]; Myanmar: T. Krajangdara & P.N. Psomadakis, pers. comm., 2015.

Generally, the taxonomy and biology of *Bythaelurus* species are poorly known. So far, studies on the biology and distribution are restricted to few species, i.e. *B*. *canescens*, *B*. *dawsoni*, *B*. *hispidus* and *B*. *lutarius*, and partially based only on a small number of specimens examined [18–21,42,44,48,53,54,60]. Therefore, more specimens are needed of several species, especially of those from the Indian Ocean and *B*. *immaculatus*. In order to further improve the knowledge of *Bythaelurus* species in this area, a comprehensive redescription of *B*. *hispidus* is currently in preparation.

### Key to the valid species of *Bythaelurus*

The present generic key has been modified from Weigmann *et al*. [4] and Weigmann & Kaschner [5]:

**1a.** Coloration variegated, with irregularly distributed spots and blotches; markings not symmetrical .............................................................................................. **2**.
**1b.** Coloration pale, dusky, or with line of pale spots; usually without conspicuous blotches but 8–9 dark, transverse bars reported for *B*. *vivaldii* ............................................... **3**.
**2a**. Grayish with a pattern of dark brown saddle-like markings on back and tail, and with variegated dark brown blotches on flanks; inside of mouth grayish with brown speckles; ventral surface whitish ....................... *B*. *clevai* (southwestern Indian Ocean: Madagascar)
**2b**. Chocolate brown dorsally with pale spots, the largest being about equal in size to eye diameter above midline, smaller below; posterior margin of dorsal, pectoral, and pelvic fins pale; ventral surface pale . . . .. . . . . . *B*. *giddingsi* (southeastern Pacific Ocean: Galapagos Islands)
**3a**. Coloration blackish with hoary gray surface and some fins white-tipped posteriorly; teeth with cusps and lateral cusps of subequal length; validity and generic assignment uncertain ............ .................................... *B*. *alcockii* (northwestern Indian Ocean: Arabian Sea)
**3b**. Coloration lighter; at least anterolateral teeth in upper jaw with median cusp much longer than lateral cusps ... ...................................................................................... **4**.
**4a**. Anal-fin base more than 1.5 times second dorsal-fin base length; color light to medium brown above with dusky areas near the fins or indistinct dark blotches, whitish to light brown below (except for *B*. *vivaldii*); maturity size 22–39 cm TL; maximum size 30–44 cm TL .............. . . .. . . .. . . .. . . .. . . .. . . .. . . .. . . .. . . .. . . .. . . .. . . .. . . .. . . .. . . .. . . .. . . .. . . .. . . .. . . .. . . .. . . .. . . .. . . .. . . .. . . .. . . .. . . .. . . .. . . ........ **5**.
**4b**. Anal-fin base equal to or less than 1.5 times second dorsal-fin base length; coloration variable but without dusky areas near fins or indistinct dark blotches; maturity size 32–59 cm TL; maximum size 42–76 cm TL ................................................... . . .................. **9**.
**5a**. Tongue and roof of mouth without (or with rudimentary) papillae; coloration largely plain with dusky areas near fins .............. *B*. *lutarius* (southwestern Indian Ocean: Mozambique)
**5b**. Tongue and roof of mouth with numerous papillae; trunk and tail with 5–6 indistinct, dark blotches (except for *B*. *vivaldii*) ............................................................... ... **6**.
**6a**. Head narrow and without distinct lateral indention anterior to outer nostrils; adult claspers broad, base width ~2% TL ............. . . .. . . ..... *B*. *tenuicephalus* (western Indian Ocean: Tanzania and Mozambique)
**6b**. Head with distinct lateral indention anterior to outer nostrils; adult claspers (if known) slender or moderately broad, base width 1.4–1.9% TL . . . .. . . .. . . .. . . .. . . .. . . .. . . .. . . .. . . .. . . .. . . .. . . .. . . .. . . . . .. **7**.
**7a**. Caudal fin short, length ~24% TL; body stout in large specimens; tongue with few papillae; reported to have 8–9 dark, broad but inconspicuous transverse bars on the back, but preserved coloration plain beige with scattered remains of dark brown ..................................................... *B*. *vivaldii* (northwestern Indian Ocean: Somalia)
**7b**. Caudal fin long, length 26.8–35.7% TL; body slender in all maturity stages; tongue densely-set with numerous papillae; trunk and tail with 5–6 indistinct, dark blotches .............................................................................................................. **8**.
**8a**. Ventral head usually uniformly yellowish or whitish; dermal denticles densely set, their surface structured by reticulations only in basal fourth; claspers abruptly narrowing before tip with knob-like apex; 8–10 spiral valve turns; adult males 27–34 cm TL (usually to ~30 cm TL) .................................. . . . *B*. *hispidus* (western and eastern Indian Ocean: Kenya to Oman, southern India and Andaman Sea)
**8b**. Ventral head usually with dark mottling; branchial, trunk and lateral caudal-fin dermal denticles loosely set, even in adults, their surface very strongly and fully structured by reticulations; claspers gradually narrowing to bluntly pointed tip without knob-like apex; 11–12 spiral valve turns; adult males 39–44 cm TL ............................. ***B*. *stewarti* n. sp.** (northwestern Indian Ocean: Error Seamount)
**9a**. Labial furrows reduced, with uppers and lowers about equal in length .................... **10**.
**9b**. Labial furrows distinct, with lowers noticeably longer than uppers ........................ **11**.
**10a**. Pre-vent length 1.3 times in tail length; preorbital snout length subequal to eye length; length of lateral trunk denticles less than twice their width ..................... *B*. *immaculatus* (western central Pacific Ocean: South China Sea)
**10b**. Pre-vent length exceeds tail length; preorbital snout length 1.3 times eye length; length of lateral trunk denticles more than twice their width ................... . . ..... *B*. *incanus* (southeastern Indian Ocean: northwestern Australia)
**11a**. Light brown to gray on dorsal and lateral surfaces with a line of white spots on sides of small individuals, whitish ventrally, fin tips with broad white areas, caudal fin also with dark bands ............................................ *B*. *dawsoni* (western Pacific Ocean: New Zealand)
**11b**. Flanks of body lack a line of white spots, ventrally darker, fin tips without broad white areas, caudal fin without dark bands ................................................................ **12**.
**12a**. Color plain dark brown or blackish above and below; maturity size 52–59 cm TL, maximum size 73 cm TL ............................................. ... *B*. *canescens* (southeastern Pacific Ocean: Peru and Chile, to Straits of Magellan)
**12b**. Coloration lighter; maturity size 36–48 cm TL, maximum size 45–55 cm TL; known only from the southwestern Indian Ocean ............................................................ **13**.
**13a**. Coloration medium to dark brown with light fin edges and a distinct dark dusky-colored snout; dorsal caudal-fin margin with prominent crest of comb-like dermal denticles; tongue and roof of mouth without papillae ...................... . . .. . . . . .... ....... *B*. *naylori* (southwestern Indian Ocean: Southwest Indian Ridge)
**13b**. Coloration plain beige to light grayish-brown; dorsal caudal-fin margin lacks prominent crest of comb-like dermal denticles; tongue and roof of mouth with numerous, partially composite papillae .................... *B*. *bachi* (southwestern Indian Ocean: Madagascar Ridge)

### Comparative material examined

#### *Bythaelurus bachi* (44 specimens)

**ZMH 26160** (holotype), adult male, 422 mm TL fresh, 390.5 mm TL 70% ethanol preserved, RV ‘Vityaz’, cruise 17, station 2707, Walters Shoals, 33°01.8’ S, 44°23.6’ E– 32°59.8’ S, 44°24.4’ E, 910–925 m depth, 19.4 m shrimp trawl, trawl # 60, on the bottom for 60 minutes, 15 Dec 1988. (Paratypes, n = 43): **ZMH 26161**, adult female with one egg case in each uterus, 405 mm TL fresh, 395 mm TL 70% ethanol preserved, RV ‘Vityaz’, cruise 17, station 2668, Walters Shoals, 33°01.2’ S, 44°36.8’ E– 33°05.2’ S, 44°39.2’ E, 1010 m depth, 19.4 m shrimp trawl, trawl # 48, on the bottom for 61 minutes, 08 Dec 1988; **ZMH 26162**, female post-embryo, 122 mm TL fresh, 120 mm TL 70% ethanol preserved, data the same as ZMH 26161; **ZMH 26163**, juvenile female, 227 mm TL fresh, 220 mm TL 70% ethanol preserved, data the same as ZMH 26161; **ZMH 26164**, juvenile female, 361 mm TL fresh, 355 mm TL 70% ethanol preserved, data the same as ZMH 26161; **ZMH 26165**, juvenile male, 210 mm TL fresh, 205 mm TL 70% ethanol preserved, data the same as ZMH 26161; **ZMH 26166**, juvenile male, 248 mm TL fresh, 240 mm TL 70% ethanol preserved, data the same as ZMH 26161; **ZMH 26167**, juvenile male, 253 mm TL fresh, 246 mm TL 70% ethanol preserved, data the same as ZMH 26161; **ZMH 26168**, juvenile male, 323 mm TL fresh, 317 mm TL 70% ethanol preserved, data the same as ZMH 26161; **ZMH 26169**, juvenile female, 290 mm TL fresh, 284 mm TL 70% ethanol preserved, RV ‘Vityaz’, cruise 17, station 2670, Walters Shoals, 33°01.6’ S, 44°49.2’ E– 33°04’ S, 44°49.1’ E, 1090–1100 m depth, 19.4 m shrimp trawl, trawl # 49, on the bottom for 60 minutes, 09 Dec 1988; **ZMH 26170**, juvenile female 292 mm TL fresh, 289 mm TL 70% ethanol preserved, data the same as ZMH 26169; **ZMH 26171**, juvenile female 295 mm TL fresh, 290 mm TL 70% ethanol preserved, data the same as ZMH 26169; **ZMH 26172**, juvenile female 314 mm TL fresh, 309 mm TL 70% ethanol preserved, data the same as ZMH 26169; **ZMH 26173**, juvenile female 320 mm TL fresh, 312 mm TL 70% ethanol preserved, data the same as ZMH 26169; **ZMH 26174**, juvenile female 339 mm TL fresh, 331 mm TL 70% ethanol preserved, data the same as ZMH 26169; **ZMH 26175**, juvenile female 346 mm TL fresh, 339 mm TL 70% ethanol preserved, data the same as ZMH 26169; **ZMH 26176**, juvenile female 350 mm TL fresh, 347 mm TL 70% ethanol preserved, data the same as ZMH 26169; **ZMH 26177**, juvenile male 254 mm TL fresh, 248 mm TL 70% ethanol preserved, data the same as ZMH 26169; **ZMH 26178**, juvenile male 305 mm TL fresh, 297 mm TL 70% ethanol preserved, data the same as ZMH 26169; **ZMH 26179**, juvenile male 314 mm TL fresh, 309 mm TL 70% ethanol preserved, data the same as ZMH 26169; **ZMH 26180**, juvenile male 322 mm TL fresh, 313 mm TL 70% ethanol preserved, data the same as ZMH 26169; **ZMH 26181**, juvenile male 328 mm TL fresh, 322 mm TL 70% ethanol preserved, data the same as ZMH 26169; **ZMH 26182**, juvenile male 354 mm TL fresh, 346 mm TL 70% ethanol preserved, data the same as ZMH 26169; **ZMH 26183**, juvenile male 359 mm TL fresh, 349 mm TL 70% ethanol preserved, data the same as ZMH 26169; **ZMH 26184**, juvenile female, 312 mm TL fresh, 297 mm TL 70% ethanol preserved, RV ‘Vityaz’, cruise 17, station 2671, Walters Shoals, 32°56’ S, 45°01’ E– 32°59’ S, 45°03’ E, 1175–1200 m depth, 19.4 m shrimp trawl, trawl # 50, on the bottom for 60 minutes, 09 Dec 1988; **ZMH 26185**, female post-embryo, 144 mm TL fresh, 132 mm TL 70% ethanol preserved, RV ‘Vityaz’, cruise 17, station 2706, Walters Shoals, 33°01’ S, 44°30’ E– 33°05’ S, 44°32’ E, 970–980 m depth, 19.4 m shrimp trawl, trawl # 59, on the bottom for 40 minutes, 15 Dec 1988; **ZMH 26186**, juvenile male, 376 mm TL fresh, 350 mm TL 70% ethanol preserved, data the same as holotype ZMH 26160; **ZMH 26187**, adult male 410 mm TL fresh, 378 mm TL 70% ethanol preserved, data the same as holotype ZMH 26160; **ZMH 26188**, adult female, 404 mm TL fresh, 393 mm TL 70% ethanol preserved, RV ‘Vityaz’, cruise 17, station 2735, Walters Shoals, 33°36’ S, 44°32’ E– 33°38’ S, 44°34’ E, 930–950 m depth, 29 m shrimp trawl, trawl # 68, on the bottom for 75 minutes, 19 Dec 1988; **ZMH 26189**, adult female 407 mm TL fresh, 400 mm TL 70% ethanol preserved, data the same as ZMH 26188; **ZMH 26190**, female post-embryo, 132 mm TL fresh, 125 mm TL 70% ethanol preserved, data the same as ZMH 26188; **ZMH 26191**, juvenile female, 265 mm TL fresh, 245 mm TL 70% ethanol preserved, RV ‘Vityaz’, cruise 17, station 2736, Walters Shoals, 33°58.1’ S, 45°01’ E– 33°57’ S, 45°02.5’ E, 1030–1050 m depth, 29 m shrimp trawl, trawl # 69, on the bottom for 47 minutes, 19 Dec 1988; **CAS 241442**, 3 specimens: adult male, 400 mm TL (tissue accession GN 15514), adult females, 422 and 423 mm TL, Walters Shoals, 34°01’ S, 45°36’ E, 950–1340 m depth, bottom trawl, 16 May 2014; **CAS 241443**, pregnant female, 405 mm TL, Walters Shoals, 34°01’ S, 45°36’ E, 950–1345 m depth, bottom trawl, 14 Apr 2014; **CAS 241444**, 2 specimens: adult female, 392 mm TL (tissue accession GN 15516), adult female, 445 mm TL (tissue accession GN 15517), Walters Shoals, 34°01’ S, 45°36’ E, 960–1210 m depth, bottom trawl, 14 Apr 2014; **CAS 241445** (tissue accession GN 12074), adult female, 404 mm TL, Walters Shoals, 34°24’ S, 45°06’ E, 1123–1294 m depth, bottom trawl, 4 May 2012; **CAS 241478** (tissue accession GN 15515), pregnant female, 408 mm TL, Walters Shoals, 34°01’ S, 45°36’ E, 950–1365 m depth, bottom trawl, 16 May 2014; **SAIAB 202736**, adult female, 403 mm TL, data the same as CAS 241443; **SAIAB 202737**, adult female, 415 mm TL, data the same as CAS 241444; **USNM 438923**, adult female, 409 mm TL, data the same as CAS 241442; **USNM 438924**, adult female, 412 mm TL, data the same as CAS 241442.

#### *Bythaelurus canescens* (Günther) (1 specimen; photographs, morphometrics, meristics, and description notes)

**BMNH 1887.12.7.1** (holotype), juvenile female, 282 mm TL, RV ‘Challenger’, station 310, off Chile, 400 fms (732 m) depth. According to Tizard *et al*. [17], station details are as follows: date 10 Jan 1876, 51°27'30" S, 74°03' W, bottom: blue mud, temperature of the seawater: 46.5° F (8.1° C) at bottom, 50.5° F (10.3° C) at surface, specific gravity of seawater at 60° F (15.6° C), distilled water at 39° F (3.9° C) = 1: 1.02451 at bottom, 1.01910 at surface, trawled.

#### *Bythaelurus clevai* (1 specimen)

**ZMH 26071**, adult male, 400 mm TL fresh, 385 mm TL 70% ethanol preserved, RV ‘Vityaz’, cruise 17, station 2645, off SW-Madagascar, 22°20'5" S, 43°03'6" E– 22°18'5" S, 43°03'1" E, 450–500 m depth, 19.4 m shrimp trawl, trawl # 40, on the bottom for 43 minutes, 2 Dec 1988.

#### *Bythaelurus dawsoni* (Springer) (2 specimens)

**ZMH 119617** (ex ISH 5215–1979), 2 juvenile males, 275 mm TL and 182 mm TL, fishing trawler ‘Wesermünde’, station 590, off western New Zealand, 41°10' S, 170°40' E, 540–560 m depth, 250'-BT (120 mm mesh size), 17 Jul 1979.

#### *Bythaelurus hispidus* (110 specimens)

**ZMH 26248**, 70 specimens: 21 females, 207–388 mm TL in fresh state, 204–380 mm TL 70% ethanol preserved, 12 juvenile males, 179–262 mm TL in fresh state, 176–258 mm TL 70% ethanol preserved, 7 subadult males, 254–282 mm TL in fresh state, 250–278 mm TL 70% ethanol preserved, 30 adult males, 274–338 mm TL in fresh state, 269–335 mm TL 70% ethanol preserved, RV ‘Vityaz’, cruise 17, station 2560, off Socotra Islands, 12°16’6” N 53°08’2” E– 12°14’7” N 53°06’2” E, 375–380 m depth, 29 m shrimp trawl, trawl # 2, on the bottom for 45 minutes, 27 Oct 1988; **ZMH 26249**, 20 specimens: 5 females, 264–357 mm TL in fresh state, 260–348 mm TL 70% ethanol preserved, 2 juvenile males, 265–266 mm TL in fresh state, 260–261 mm TL 70% ethanol preserved, 3 subadult males, 268–272 mm TL in fresh state, 262–273 mm TL 70% ethanol preserved, 10 adult males, 282–324 mm TL in fresh state, 276–318 mm TL 70% ethanol preserved, RV ‘Vityaz’, cruise 17, station 2566, off Socotra Islands, 12°17’5” N, 53°09’ E– 12°14’5” N, 53°06’5” E, 384–390 m depth, 30 m bottom trawl, trawl # 6, on the bottom for 58 minutes, 28 Oct 1988 (taken together with 6 further specimens: 2 juvenile and 4 adult males, which were not retained); **ZMH 26250**, 10 specimens: 2 adult females, 332 and 364 mm TL in fresh state, 325 and 349 mm TL 70% ethanol preserved, 1 gravid female, 346 mm TL in fresh state, 338 mm TL 70% ethanol preserved, with one female and one male embryo, 121.6 and 126.7 mm TL 70% ethanol preserved, respectively, 2 juvenile males, 166–237 mm TL in fresh state, 156–232 mm TL 70% ethanol preserved, 1 subadult male, 285 mm TL in fresh state, 280 mm TL 70% ethanol preserved, 2 adult males, 324–329 mm TL in fresh state, 317–326 mm TL 70% ethanol preserved, RV ‘Vityaz’, cruise 17, station 2830, off Socotra Islands, 12°14’8” N, 53°06’2” E– 12°17’8” N, 53°08’9” E, 395–420 m depth, 29 m shrimp trawl, trawl # 101, on the bottom for 80 minutes, 16 Jan 1989 (taken together with 417 further specimens, which were not retained). All 100 preserved and 423 discarded specimens were collected by M.F.W. Stehmann. **SMF 11465**, subadult male, 212.9 mm TL and juvenile male 176.6 mm TL, Gulf of Mannar, 457 m depth, 08 April 1970; **BMNH 1898.7.13.21**, adult male, 258.5 mm TL, off Andaman Islands, 338 m depth, from the Indian Museum, Calcutta; **SAIAB 13741** (field no. 80–20) adult male, 318 mm TL, off Kenya, 03°26’ S, 40°23’ E, 484 m depth, 11 Dec 1980 (photographs and radiographs); **SAIAB 14057** (field no. PCH 80–19), female, 252 mm TL, off Kenya, 03°49’ S, 40° E, 11 Dec 1980 (photographs and radiographs); **SAIAB 14103** (field no. PCH 80–24), adult male, 310 mm TL, off Kenya, 03°11’ S, 40°38’ E, 12 Dec 1980 (photographs and radiographs); **USNM 221384**, female, 285 mm TL, adult male, 260 mm TL, and juvenile male, 194 mm TL, RV ‘Anton Bruun’, cruise 1, station 23, off Andaman Islands, 10°39’ N, 96°35’ E, 367–375 m depth, 40 ft shrimp trawl, 24 Mar 1963 (photographs only); **ZSI 13120** (holotype), juvenile male, 222 mm TL, station 115, off Andaman Islands, 11°31'40" N, 92°46'40 "E, 344–402 m depth, Dec 1890 (photographs only).

#### *Bythaelurus lutarius* (Springer & D’Aubrey) (5 specimens)

**USNM 205135** (holotype), adult male, 323 mm TL, RV ‘Anton Bruun’, cruise 8, station 396B, 25°32' S, 33°24' E, 450–455 m depth, shrimp trawl, 28 Sep 1964 (photographs and morphometrics plus meristics); **USNM 221660** (paratype, field no. 182), female, 323 mm TL, data the same as holotype (photographs only); **SAIAB 6141**, three adult females, 340–370 mm TL, caught off southern Mozambique, 10 Nov 2000 (photographs and radiographs).

#### *Bythaelurus naylori* (21 specimens; photographs, morphometrics and meristics)

**CAS 237869** (holotype), adult male, 452 mm TL, Southwest Indian Ridge, 35°10’ S, 53°40’ E, 800–1300 m depth, bottom trawl, collected by P.J. Clerkin, 21 May 2014. **(**Paratypes, n = 15): **CAS 237870** (GN 123330), adult male, 455 mm TL, Southwest Indian Ridge, 33°55’ S, 55°16’ E, 1008–1190 m depth, midwater trawl, collected by P.J. Clerkin, 28 Apr 2014; **CAS 237872**, female, 506 mm TL, Southwest Indian Ridge, 34°53’ S, 54°23’ E, 811–1150 m depth, bottom trawl, collected by P.J. Clerkin, 28 Apr 2014; **CAS 237873**, female, 505 mm TL, Southwest Indian Ridge, 36°49’ S, 52°05’ E, 1438–1443 m depth, bottom trawl, collected by P.J. Clerkin, 21 May 2014; **CAS 237941**, adult male, 511 mm TL, Southwest Indian Ridge, 35°08’ S, 53°42’ E, 861–1160 m depth, bottom trawl, collected by Ryan Downie, 11 Mar 2014; **CAS 237942** (GN 11840), female, 548 mm TL, Southwest Indian Ridge, 39°00’ S, 46°30’ E, 800–1200 m depth, bottom trawl, collected by P.J. Clerkin, 17 Mar 2012; **CAS 237943** (GN 11841), female, 450 mm TL, Southwest Indian Ridge, 39°00’ S, 46°30’ E, 800–1200 m depth, bottom trawl, collected by P.J. Clerkin, 17 Mar 2012; **CAS 237944** (GN 11857), adult male, 491 mm TL, Southwest Indian Ridge, 35°10’ S, 53°40’ E, 800–1300 m depth, bottom trawl, collected by P.J. Clerkin, 8 Apr 2012; **CAS 238013** (GN 11866), adult male, 502 mm TL, Southwest Indian Ridge, 35°10’ S, 53°40’ E, 800–1300 m depth, bottom trawl, collected by P.J. Clerkin, 8 Apr 2012; **CAS 238070** (GN 12399), immature male, 341 mm TL, Southwest Indian Ridge, 33°55’ S, 55°16’ E, 1040–1250 m depth, bottom trawl, collected by P.J. Clerkin, 29 Apr 2014; **CAS 238071** (GN 12378), female, 301 mm TL, Southwest Indian Ridge, 33°55’ S, 55°16’ E, 1008–1185 m depth, bottom trawl, collected by P.J. Clerkin, 29 Apr 2014; **SAIAB 200728**, adult male, 482 mm TL, Southwest Indian Ridge, 35°08’ S, 53°42’ E, 861–1160 m depth, bottom trawl, collected by Ryan Downie, 11 Mar 2014; **SAIAB 200729**, female, 464 mm TL, Southwest Indian Ridge, 33°55’ S, 55°16’ E, 1008–1190 m depth, midwater trawl, collected by P.J. Clerkin, 28 Apr 2014; **iSAM MB-F041239** (GN 12347), female, 453 mm TL, Southwest Indian Ridge, 35°08’ S, 53°42’ E, 89–1240 m depth, bottom trawl, collected by P.J. Clerkin, 30 Apr 2014; **USNM 432400** (GN 12371), adult male, 465 mm TL, Southwest Indian Ridge, 35°08’ S, 53°42’ E, 752–780 m depth, bottom trawl, collected by P.J. Clerkin, 23 Apr 2014; **USNM 432401**, female, 466 mm TL, Southwest Indian Ridge, 35°08’ S, 53°42’ E, 861–1160 m depth, bottom trawl, collected by Ryan Downie, 11 Mar 2014. (Non-types, n = 5): **CAS 243692**, immature male, 316 mm TL, data the same as CAS 238071; **CAS 243693**, female, 395 mm TL, data the same as USNM 432400; **CAS 243695**, female, 483 mm TL, data the same as iSAM MB-F041239; **CAS 243696**, subadult male, 402 mm TL, Southwest Indian Ridge, 34° S, 55°15’ E, 850–1075 m depth, bottom trawl, collected by P.J. Clerkin, 24 May 2014; **CAS 243697**, immature male, 270 mm TL, Southwest Indian Ridge, 35°08’ S, 53°42’ E, 880–1200 m depth, bottom trawl, collected by P.J. Clerkin, 23 Apr 2014.

#### *Bythaelurus tenuicephalus* (2 specimens)

**ZMH 10163** (holotype), adult male, 279 mm TL, RV ‘Valdivia’, station 245, off North Tanzania, 5°27'9" S, 39°18'8" E, 463 m depth, 22 March 1899; **ZMH 26070** (paratype), juvenile male, 291 mm TL fresh, 285 mm TL 70% ethanol preserved, RV ‘Vityaz’, cruise 17, station 2622, off South Mozambique, 21°12'8" S, 35°41'8" E– 21°16'6" S, 35°41'3" E, 490–550 m depth, 29 m shrimp trawl, trawl # 25, on the bottom for 63 minutes, 21 Nov 1988.

#### *Bythaelurus vivaldii* (2 specimens)

**ZMB 17410** (holotype), presumably adult female, 325 mm TL, RV ‘Valdivia’, station 265, off Somalia, 6°24'1" N, 49°31'6" E, 628 m depth, 30 Mar 1899; **ZMB 22423** (paratype), presumably immature female, 272.7 mm TL, data the same as holotype ZMB 17410.

#### *Halaelurus quagga* (Alcock) (2 specimens)

**ZMH 103101** (ex ISH 182–1965), adult male, 286 mm TL, RV ‘Meteor’, Indian Ocean Expedition, station 189, off Southwest India, 09°40’ N, 75°38.8’ E, 138–210 m depth, otter trawl, 10 Feb 1965; **ZMH 103223** (ex ISH 161–1965), adult male, 277 mm TL, RV ‘Meteor’, Indian Ocean Expedition, station 78, off Djibouti, 12°25’ N, 43°28.8’ E, 185 m depth, Agassiz trawl, 12 Dec 1964.
